# Development and validation of the OH-KAP survey for use with pastoral and other rural communities in Africa

**DOI:** 10.1186/s42522-026-00213-8

**Published:** 2026-05-22

**Authors:** Farah I. Mumin, Siobhan M. Mor

**Affiliations:** 1 Institute of Infection, Veterinary and Ecological Sciences, University of Liverpool, Neston, UK; 2 International Livestock Research Institute, Nairobi, Kenya; 3 Red Sea University, Bosaso, Puntland State Somalia

**Keywords:** One health, Knowledge, Attitudes, Practices (KAP), OH-KAP, Pastoral, Item response theory (IRT), Validation

## Abstract

**Background:**

Evaluating One Health at community-level requires robust, valid measures of what communities know, believe, and do about health risks shared by people, animals, and the environment. This study aimed to develop and validate a One Health Knowledge, Attitudes and Practices (OH-KAP) instrument tailored to (agro-)pastoralist and mixed-farming systems in Africa, covering key One Health topics including zoonoses, antimicrobial resistance, and food safety.

**Methods:**

An initial pool of 156 items was derived from the literature and refined through expert content validation to 126 items. Subsequently, the questionnaire was translated into Somali and field-tested with 300 adults in Middle Shabelle, Somalia. Psychometric analysis of knowledge and attitudes items was undertaken using classical test theory, exploratory factor analysis, and bifactor item response theory (2-parameter logistic models for binary knowledge items; graded response models for Likert-scale attitudes items). Practice items were analysed using exploratory graph analysis and community detection.

**Results:**

The final instrument included 27 knowledge, 19 attitude and 29 practice items. The knowledge sub-scale loaded onto a general One Health factor with 4 domain-specific subfactors: zoonotic transmission and environmental risks; animal bites and safe food handling; AMR; and direct contact and food contamination risks (reliability: α = 0.94; model fit: CFI = 0.96, RMSEA 0.08). The attitudes sub-domain loaded onto a general One Health factor with 5 domain-specific subfactors: hand hygiene; animal husbandry; zoonotic outbreaks; AMR; and antimicrobial use (AMU) (α = 0.93; CFI = 0.99, RMSEA 0.05). Practices clustered into four stable domains: animal management and AMU; direct contact and exposure control; responsible husbandry, food safety and stewardship; and hand hygiene and disease reporting (bootstrap stability > 65%).

**Conclusions:**

The developed OH-KAP is a concise, field-ready tool for quantifying integrated One Health knowledge, attitudes and practices. It supports baseline assessments and monitoring associated with awareness-creation and behaviour-change programming. Future work should extend coverage to additional One Health priorities and assess invariance across settings and languages.

**Supplementary Information:**

The online version contains supplementary material available at 10.1186/s42522-026-00213-8.

## Introduction

One Health is an integrative approach that recognizes the interdependence of human, animal, and environmental health and calls for collaborative, cross-sectoral strategies to address shared health risks [2]. The approach has been endorsed globally as a critical tool for addressing complex health challenges [61]. While One Health offers societal benefits across the spectrum, operationalizing this approach is particularly important in rural communities in Africa, where close contact between people, animals, and the environment shape daily life. Pastoral, agro-pastoral, and mixed crop-livestock farming communities represent a substantial proportion of rural populations in sub-Saharan Africa. Pastoralists are defined by their reliance on extensive livestock keeping as a primary livelihood, often involving seasonal mobility and communal grazing. Agro-pastoralists combine crop cultivation with livestock rearing, while mixed farmers integrate both in more sedentary systems [58]. These communities frequently operate in contexts characterised by high livestock-human-environment contact, limited access to formal health services, low formal literacy rates, and informal healthcare-seeking behaviours, all factors that increase vulnerability to a range of One Health hazards [12, 47].

Among the most significant of these hazards are zoonoses, AMR and food safety. Frequent animal-human interaction in these contexts increases vulnerability to zoonotic diseases through direct exposure to animals and shared environments [6, 45]. Food safety hazards emerge as a critical concern, driven by local practices in food handling, storage, and preparation, alongside environmental challenges including limited access to clean water and sanitation [36, 46, 52]. Antimicrobial resistance (AMR) presents a distinct but related threat, shaped by limited veterinary and healthcare infrastructure, inadequate regulatory frameworks, and prevalent informal antimicrobial use [17, 28]. Understanding how rural communities perceive and respond to such threats is essential for designing targeted interventions, strengthening public health systems, and supporting sustained behavior change [27].

The WHO Quadripartite One Health Joint Plan of Action (2022–2026) and a recent report from the Lancet One Health Commission [48, 60] both highlight the importance of community engagement in operationalizing One Health. Both advocate for evidence-informed, locally-driven approaches that incorporate community knowledge and behaviors into zoonotic disease prevention and control, amongst other areas. However, there is currently a critical gap in validated tools to measure community knowledge, attitudes, and practices (KAP), which hinders efforts to design and evaluate effective community-based interventions.

When rigorously developed and validated, KAP surveys can generate reliable evidence to inform health system planning and policy [57]. KAP questionnaires are psychometric diagnostic tools that are used to gather information about a set of understandings a person has (*knowledge*), their beliefs and leaning towards a particular subject (*attitude*), and an individual’s actions (*practices*) resulting from both knowledge and attitudes [8]. They are based on the Health Belief Model, which posits that behavioral change is influenced by a person’s perceptions of a health threat and their confidence in their ability to take action. Over time, these perceptions and responses can shape new behaviors that become routine [29]. Various studies suggest that one’s KAP level is linked to the ability to practice preventive measures, respond to treatments, and improve personal health [32, 41, 55]. As data collection tools, KAP surveys can rapidly and efficiently produce data that can be easily interpreted, summarized, and generalized to the wider population by training local enumerators [10, 54]. Despite these advantages, their effectiveness ultimately depends on the quality of their development and validation processes.

In practice, KAP surveys face persistent methodological challenges. Their widespread use in research and development projects does not always translate into accurate or reliable data [15, 26, 49]. Many suffer from poor design during development [37] and a lack of psychometric testing for validity and reliability [34], leading to instruments that may fail to measure what they intend to assess. Traditionally, items are treated as independent, and scores are often derived by summing item responses. This assumes all items contribute equally to the total score and does not account for the underlying traits or dimensions being measured. Inconsistent design and measurement approaches, inadequate contextual adaptation, and insufficient validation undermine interpretability and comparability of these KAP surveys across contexts [37]. These limitations have prompted growing interest in psychometric methods such as factor analysis and item response theory (IRT) to enhance structural validity and measurement precision [19].

Although structured surveys have been used to explore KAP in One Health contexts, they often focus on individual topics such as zoonoses [1], food safety [46] or AMR [53], rather than capturing One Health as a broader construct. While many recent KAP studies ask about community awareness of ‘One Health,’ they rarely articulate the concept or evaluate it as an integrated construct, and published examples of formal validation remain limited [33]. Without rigorous validation, KAP surveys may measure constructs inconsistently or inaccurately [11, 20], limiting their effectiveness in guiding One Health interventions.

This study addresses this gap by developing and validating the One Health Knowledge, Attitudes and Practices (OH-KAP) survey. OH-KAP is a psychometrically validated instrument designed to assess KAP in rural communities related to several domains typically addressed by One Health initiatives, namely: zoonotic diseases, food safety, and antimicrobial resistance (hereafter referred to as ‘One Health hazards’). Through a rigorous development process and evaluation of its reliability and construct validity, OH-KAP offers a psychometrically sound tool that can be used to assess baseline knowledge, attitudes and practices or change in these constructs associated with a One Health initiative.

## Methodology

To ensure conceptual clarity and psychometric rigor, this study followed a two-phase approach in developing the OH-KAP instrument. Phase I involved iterative item development, expert-led content validation, and field testing in pastoralist communities. Phase II applied robust validation procedures, including exploratory factor analysis and bifactor Item Response Theory (IRT) modeling for the knowledge and attitude subscales, and exploratory graph analysis (EGA) for the practice subscale. All analyses were performed in R (version 4.3.1). The R scripts for the knowledge, attitude, and practice subscales are provided as Supplementary Files 1, 2, and 3, respectively, to facilitate transparency and reproducibility.

### Phase I: Instrument development and field testing

#### Item generation

The initial items for the OH-KAP were developed based on a literature review as well as expert input from the authors which is grounded in extensive field experience in the Horn of Africa. A targeted search of the PubMed database was conducted using terms such as ‘One Health’, ‘zoonoses’, ‘food safety’, ‘AMR’, ‘knowledge’, ‘attitudes’, and ‘practices’ to identify potential items to include in content validation. In addition, we reviewed WHO and WOAH guidelines [40, 62] which provided a practical framework for KAP survey construction, including ensuring clarity and providing guidance on structuring questions by domain. An initial draft questionnaire was developed, comprising 48 knowledge items (binary responses), 48 attitude items (Likert-scale responses), and 60 practice items (mixed response types). Internal expert review by the authors (FIM and SM) resulted in further refinement of item wording given the intended use of the questionnaire with pastoralists, agro-pastoralists and mixed farmers in the Horn of Africa.

#### Content validation

Content validity of the OH-KAP instrument was evaluated using the Content Validity Index (CVI) method [63]. A panel of eight subject matter experts independently rated the relevance of each item in measuring One Health-related knowledge, attitudes, and practices on a 4-point Likert scale (1 = Not Relevant to 4 = Highly Relevant). The panel included professionals with diverse expertise (veterinary medicine, public health, epidemiology, One Health, environmental health, and food safety), working in different sectors (academic institutions, international research organizations, national public health systems and NGOs) across the Horn of Africa (Ethiopia, Kenya, Somalia) and Europe. Scale-level content validity was assessed using the average method (S-CVI/Ave), providing an indication of overall expert agreement on item relevance. Item-level CVIs (I-CVI) were calculated as the proportion of experts who rated each item as 3 or 4. Items with I-CVI ≥ 0.83 were retained consistent with established practices [39] while items with low I-CVI scores or those deemed redundant or overlapping were flagged for potential removal prior to field testing.

#### Field testing

##### Digitization of the questionnaire

Retained items were translated, digitalized using KoboToolbox [35], and administered via the KoboCollect mobile application. The digital form included both English and Somali translations and incorporated skip logic and validation checks to improve data quality and to ensure consistency during administration. Retained items were translated into written Somali by FIM, a native Somali speaker with extensive experience conducting field research in pastoral communities in the Horn of Africa. The translation process attended to both linguistic accuracy and cultural appropriateness, including the use of terminology familiar to participants with limited formal literacy. While no formal back-translation procedure was undertaken, the Somali translation was reviewed in practice during enumerator training, where six locally recruited enumerators themselves native Somali speakers read all items aloud and flagged any phrasing that was unclear or unnatural. This provided a practical check on the translation prior to field deployment. We acknowledge that formal cross-cultural adaptation and back-translation remain important priorities for future work extending the instrument to other languages and settings.

##### Sampling strategy

The questionnaire was administered to 300 participants (100 pastoralists, 100 agro-pastoralists, 100 mixed farmers) from the Middle Shabelle region, Somalia. Eligible participants were men or women aged over 18 years and residing in the region at the time of interview. There is no universally agreed-upon sample size for validation [59]; prevailing recommendations suggest 5–10 participants per item, with smaller ratios of around 3–5 considered acceptable when items have high communalities and stable factor structures [7, 21]. Our ratio of 2.4 people per item is slightly below these lower benchmarks; however, smaller samples can still yield valid results when dimensionality is clear and robust estimation methods are applied [18] as was the case in this study. Although the sample was drawn from one region of Somalia, the instrument was designed for use across pastoral and rural communities in Africa, and external validation in additional settings is encouraged.

##### Interview procedure

All interviews were conducted face-to-face in Somali, following written informed consent, with written signatures substituted with thumbprint in cases of illiteracy. Enumerators read each item aloud from the digital form without interpretation or elaboration to minimize interviewer bias and ensure standardized delivery of items using the Somali translation.

##### Enumerator training and bias reduction

A team of six enumerators, representing a mix of human and animal health expertise, received structured training covering the study objectives, ethical research conduct, standardized interviewing techniques, and digital data entry using KoboCollect. The training emphasized the importance of neutrality, confidentiality, and consistent translation of items. Field supervision was provided by FIM, who conducted regular spot checks and monitored daily uploads to the Kobo server to ensure data quality and adherence to the protocol.

### Phase II: Psychometric validation

#### Knowledge subscale

##### Data preparation

Data cleaning was undertaken prior to analysis, and datasets were checked for completeness and consistency. For the Knowledge subscale, correct responses were predetermined based on scientific evidence and expert consensus. Participant answers matching the correct response were coded as correct (1), while both “False” and “Don’t know” were coded as incorrect (0), since each indicates the absence of accurate knowledge.

##### Internal consistency

Internal consistency was assessed using Cronbach’s alpha (α). A threshold of α ≥ 0.70 was used to indicate acceptable reliability [56].

##### Exploratory factor analysis (EFA)

Prior to conducting factor analysis, the suitability of the dataset for factor extraction was assessed. Bartlett’s Test of Sphericity was performed to verify the presence of sufficient item correlations, while the Kaiser-Meyer-Olkin (KMO) Measure of Sampling Adequacy was used to assess the appropriateness of the sample size for EFA. A significant Bartlett’s test (*p* < 0.05) [9] and KMO values above 0.70 indicated suitability for factor extraction [31].

The number of factors to retain was determined using Parallel Analysis, which compares the observed eigenvalues from the dataset to those generated from randomly simulated data [38]. Retention was further supported by the Kaiser criterion (eigenvalue > 1) [30], though parallel analysis was the primary method. EFA was conducted using the Minimum Residual (MINRES) estimation method, which is robust to non-normal data distributions [22]. An Oblimin rotation was applied to allow for correlated factors, reflecting the expected interrelatedness among One Health hazards [16]. Items were assigned to factors based on their highest standardized loading, with a minimum loading threshold of 0.40 [56]. It is important to note that no separate Confirmatory Factor Analysis (CFA) was conducted on the same sample. Instead, the bifactor IRT model was used to evaluate the EFA-informed structure at the item level, with five-fold cross-validation applied to confirm model generalizability and guard against overfitting.

#### Bifactor IRT modeling

##### Model comparison and selection

Three IRT models were evaluated for the knowledge subscale using the *mirt* package in R [13]: unidimensional, multidimensional, and bifactor. Model fit was assessed using standard indices (AIC, BIC, CFI, TLI, RMSEA, SRMSR). The bifactor model, which specified one general latent trait and multiple domain-specific factors, consistently demonstrated superior fit and interpretability.

##### Item refinement and estimation

The bifactor model was estimated using the 2PL model for binary (knowledge) items. An iterative refinement process was used to improve model fit and conceptual coherence. Items were removed based on: (a) significant local misfit (S-X², *p* < 0.05), (b) low discrimination (a < 0.40), (c) flat item information, or (d) theoretical misalignment. After each iteration, we re-conducted EFA and refitted bifactor models, systematically removing problematic items until satisfactory model fit was achieved. Final item retention decisions were grounded in both statistical indicators and theoretical interpretability.

##### Model visualization

A Sankey diagram [51] was used to show how each item loads simultaneously onto the general factor and one specific factor, reflecting the bifactor model structure. Standard IRT visualization tools, such as the plot() function in the *mirt* package, are not designed to accommodate the complex structure of bifactor models, which require the simultaneous representation of both general and specific factor loadings. To address this limitation, custom visualization was developed using base R and the ggplot2. For the knowledge subscale, Item Characteristic Curves (ICCs) and Item Information Functions (IIFs) were plotted to evaluate item performance across the general and specific factors. In addition, a Test Information Function (TIF) was plotted to assess overall measurement precision.

##### Cross-validation

To assess the stability and generalizability of the bifactor model, a 5-fold cross-validation procedure was performed [23]. The dataset was randomly partitioned into five subsets, with each subset serving once as a validation set while the remaining folds were used for training. Model performance was evaluated across folds using mean RMSEA and SRMSR for absolute fit, and CFI and TLI for structural validity. Item parameter stability was assessed by comparing discrimination (a) and difficulty (b) estimates across folds, confirming the robustness of the final models.

#### Attitudes subscale

##### Data preparation

For the Attitudes subscale, responses were recoded into three-point ordinal scales to reflect degree of endorsement. Strongly positive responses (e.g., “Strongly agree,” “Very important,” “Very concerned,” “Very risky”) were coded as 2, moderate responses (e.g., “Somewhat agree,” “Somewhat important,” “Somewhat concerned,” “Somewhat risky”) as 1, and the lowest-level responses (e.g., “Don’t agree,” “Not important,” “Not concerned,” “Not risky”), as well as “Don’t know,” were coded as 0. “Don’t know” was treated as 0 to indicate the absence of a clear belief or positive attitude toward the construct.

##### Analysis pipeline (internal consistency, EFA, IRT, validation)

Procedures for the attitude subscale followed the same analysis pipeline as the knowledge subscale above, including internal consistency assessment, EFA, bifactor IRT modelling, and five-fold cross-validation. The main methodological difference was the use of the IRT Graded Response Model (GRM), which is appropriate for modelling ordinal response categories. For the attitude subscale, OCCs and IIFs were generated to evaluate the behaviour and precision of polytomous items across the latent attitude continuum.

#### Practice subscale

##### Data preparation

All items were framed in terms of frequency of behavior and recoded into three-point ordinal scales. Responses of “Yes, always” were coded as 2, “Yes, sometimes” as 1, and “No, never” as 0. “Don’t know/Don’t want to answer” was also coded as 0, reflecting no reported engagement in the behavior. Reverse coding was applied to negatively phrased items so that, across all practice items, higher scores consistently indicated more desirable practices.

##### Exploratory graph analysis procedure

The practice subscale was analysed using EGA, a network-based method that groups items into communities based on how strongly they are related to one another [14, 25]. This approach was selected because traditional factor analysis produced an excessively high number of factors with poor interpretability, and the IRT models did not fit the data well, likely reflecting the complex and varied nature of the behaviours being measured. Before analysis, items were screened for redundancy using a procedure that detects highly overlapping questions [24]. Redundant items were removed to improve the clarity of the resulting structure. EGA was then applied to determine how many item communities were present by mapping the statistical connections between items and grouping together those that most often appeared in the same cluster.

##### Stability and interpretation

To evaluate the stability of the results, a bootstrap analysis with 500 replications was conducted. This procedure repeatedly re-analysed the data to determine whether the same communities and item assignments were recovered across samples. Items showing low stability (appearing in the same community in fewer than 50% of replications) were removed, and the analysis was repeated on the cleaned dataset. Communities were thematically labelled based on the behaviours they represented, acknowledging that some communities contained items from different thematic areas due to real-world behavioural overlap. Finally, standardized network loadings were computed for the final model to quantify item–dimension associations.

## Results

### Phase I: Instrument development and field testing

#### Content validation

**Fig. 1:**
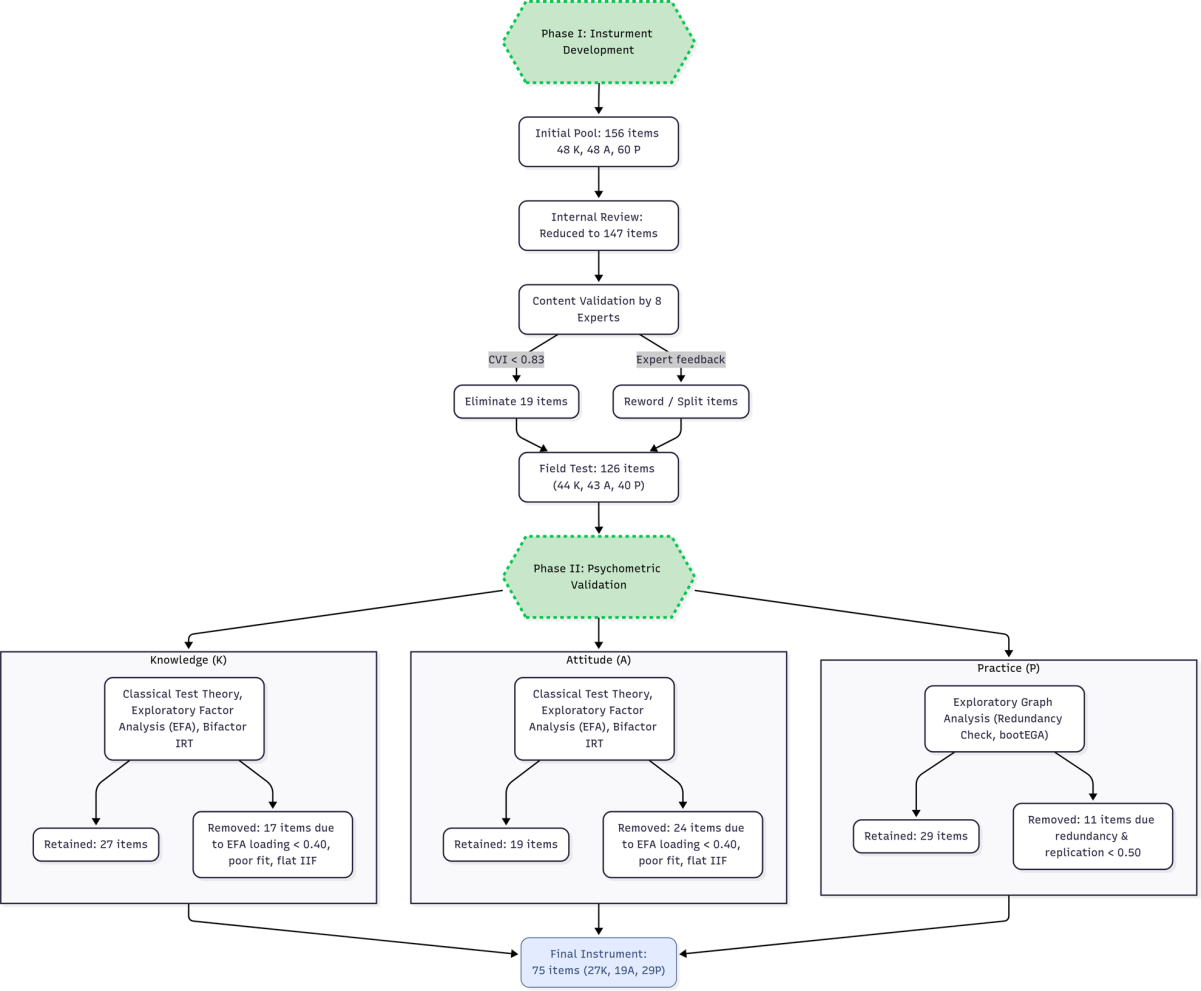
A flowchart outlining the two-phased methodology for the development and psychometric validation of the OH-KAP survey. Phase 1 included item development and content validation, while Phase 2 focused on field testing and statistical analysis to produce the final instrument. K, knowledge; A, Attitudes; P, Practices

The full list of the 147 items reviewed by the expert panel, along with their I-CVI scores, is provided in Supplementary File 4. There was strong overall agreement in the responses by experts (S-CVI/Ave = 0.903). Out of 147 items reviewed by the expert panel, the I-CVI ranged from 0.38 to 1.00. A total of 128 items (87.1%) met or exceeded the threshold of 0.83, indicating good content validity. Based on these results, and expert feedback which resulted in rewording of some items, 126 items were retained and subsequently subjected to field testing and validation (Fig. 1).

#### Sample characteristics

Of the 300 participants involved in field testing of the questionnaire, 50% were female. Most participants were aged ≤ 30 years (38.7%) or 31–45 years (37%), with fewer participants aged 46–60 years (20%), and ≥ 61 years (4.3%). Most respondents reported limited literacy: 64.7% were unable to read and write, 33.3% were literate, and 2% declined to answer. Livelihoods were evenly distributed across pastoralism, agro-pastoralism, and mixed crop-livestock farming (33.3% each). Livestock ownership was high, with 94.7% owning cattle, 88.7% goats, 79.7% sheep, 75% poultry, 60.7% equine, and 17% camels. Most participants (78%) had over 10 years of livestock-raising experience, 15.7% had 3–10 years, 0.7% had less than 3 years, and 5.7% were unsure. Only 13.7% of participants reported having previously attended any human or animal health-related training.

### Phase II: Psychometric validation

The final OH-KAP instrument included 27 knowledge items, 19 attitudes items and 29 practice items. The results below are from the psychometric validation of the final instrument items, which were selected after multiple rounds of refinement and the removal of poorly performing items (Fig. 1).

#### Knowledge subscale

##### Internal consistency

The final knowledge subscale of the OH-KAP demonstrated excellent internal consistency, with a Cronbach’s alpha of 0.94 (95% CI: 0.94–0.96), confirming high reliability and internal coherence among items.

##### EFA for knowledge subscale

**Fig. 2:**
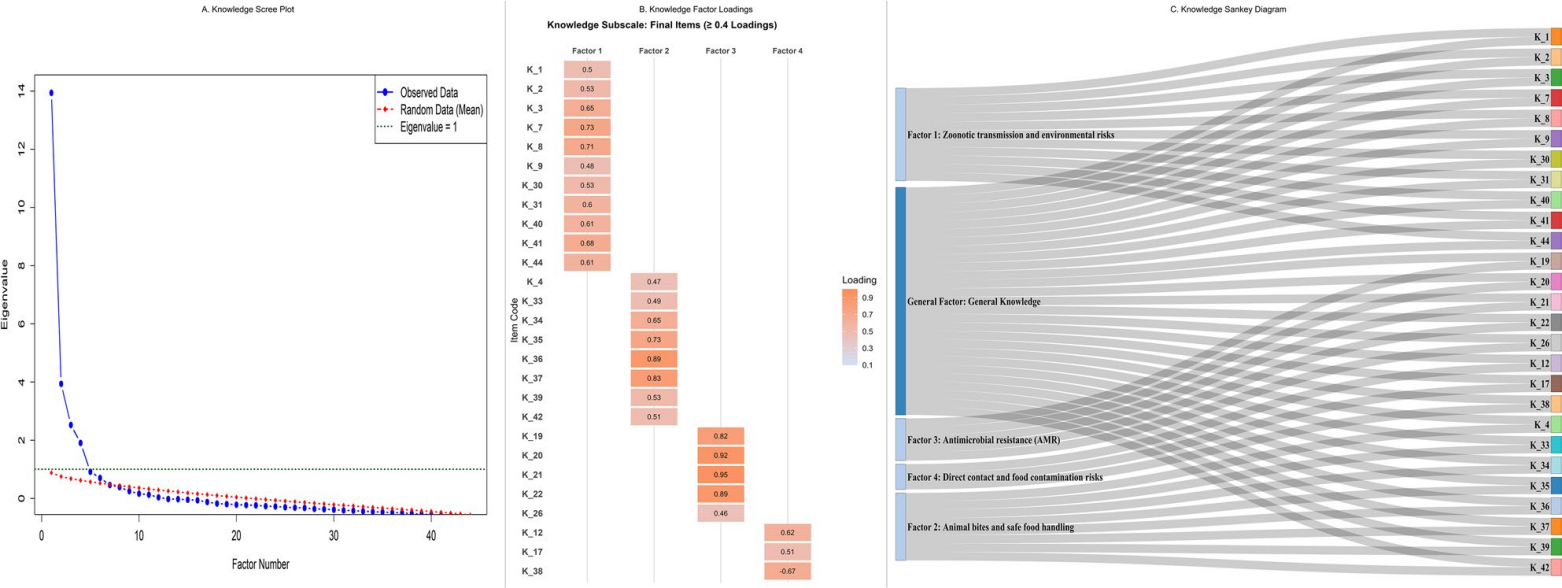
Exploratory Factor Analysis (EFA) and bifactor model results for the knowledge subscale of the OH-KAP. (**A**) Parallel analysis scree plot: The solid blue line represents observed eigenvalues from the actual data; the dashed red line shows mean eigenvalues generated from random data (parallel analysis); and the green dotted line indicates the Kaiser criterion (eigenvalue = 1). Four factors were retained based on observed eigenvalues exceeding those of the random data and interpretability of the scree plot. (**B**) Exploratory Factor Analysis (EFA) loadings: Standardized factor loadings for the final 27 retained knowledge items (loading ≥ 0.40). (**C**) Sankey diagram from bifactor IRT model: Each item loads onto both the general One Health knowledge factor (G) and one of four domain-specific factors namely Factor 1: zoonotic transmission and environmental risks, Factor 2: animal bites and safe food handling, Factor 3: antimicrobial resistance (AMR), and Factor 4: direct contact and food contamination risks. This structure reflects the multidimensional yet unified nature of the One Health knowledge construct. Note: Item codes begin with “K” to indicate knowledge items

Bartlett’s Test of Sphericity was significant (χ²(946) = 10,178.48, *p* < 0.001), and the Kaiser–Meyer–Olkin (KMO) measure was 0.908, classified as ‘marvelous’ in Kaiser’s original scale, indicating the data were highly suitable for factor analysis. Parallel analysis initially suggested six factors; however, only four were retained based on interpretability, eigenvalues > 1, and the scree plot (Fig. 2A). These four factors accounted for 46.4% of the total variance. All items had primary loadings ≥ 0.40 (Fig. 2B), and factor correlations ranged from − 0.015 to 0.398, indicating weak inter-factor associations and supporting the use of a bifactor modelling framework.

#### Bifactor IRT results

##### Model structure

The bifactor IRT model for the knowledge subscale included a general One Health knowledge construct and four domain-specific factors: zoonotic transmission and environmental risks, animal bites and safe food handling, antimicrobial resistance (AMR), and direct contact and food contamination risks (Fig. 2C). The final model retained 27 items.

##### Item discrimination and difficulty

Most knowledge items demonstrated moderate to high discrimination (a), indicating effective differentiation across participants’ knowledge of key One Health hazards — namely zoonoses, antimicrobial resistance (AMR), and food safety (Table 1). High-performing items spanned domains, including antimicrobial resistance (e.g., K_21, a = 4.6), zoonoses (e.g., K_12, a = 4.1), and food safety (e.g., K_41, a = 2.9). Only one item (K_38) had low discrimination (a < 1), suggesting overall strong psychometric quality. Item difficulty (d) values ranged widely, reflecting varying cognitive demand across items. Easier items (e.g., K_3; d = − 2.3) were likely to be endorsed even by individuals with limited knowledge in any of the domains, whereas more challenging items (e.g., K_21, d = 4.9; K_22, d = 3.9) required integrated understanding across zoonoses, AMR, and food safety, functioning as indicators of more advanced, cross-domain One Health knowledge.

##### Model fit and local dependence

Global model fit statistics supported the adequacy of the bifactor structure: M²(297) = 822.99, *p* < 0.001; RMSEA = 0.077 (90% CI: 0.071–0.083); SRMSR = 0.177; CFI = 0.964; TLI = 0.957. Most items exhibited acceptable local fit based on residual correlations and local dependence statistics (Supplementary File 5). However, a few items (e.g., K_35, K_38) showed signs of local dependence, potentially indicating shared content or response patterns. These items were retained due to their conceptual relevance and contribution to content validity. Overall, the findings support the construct validity of the knowledge subscale and the adequacy of the general factor in capturing One Health knowledge.

##### Item and test information

**Fig. 3:**
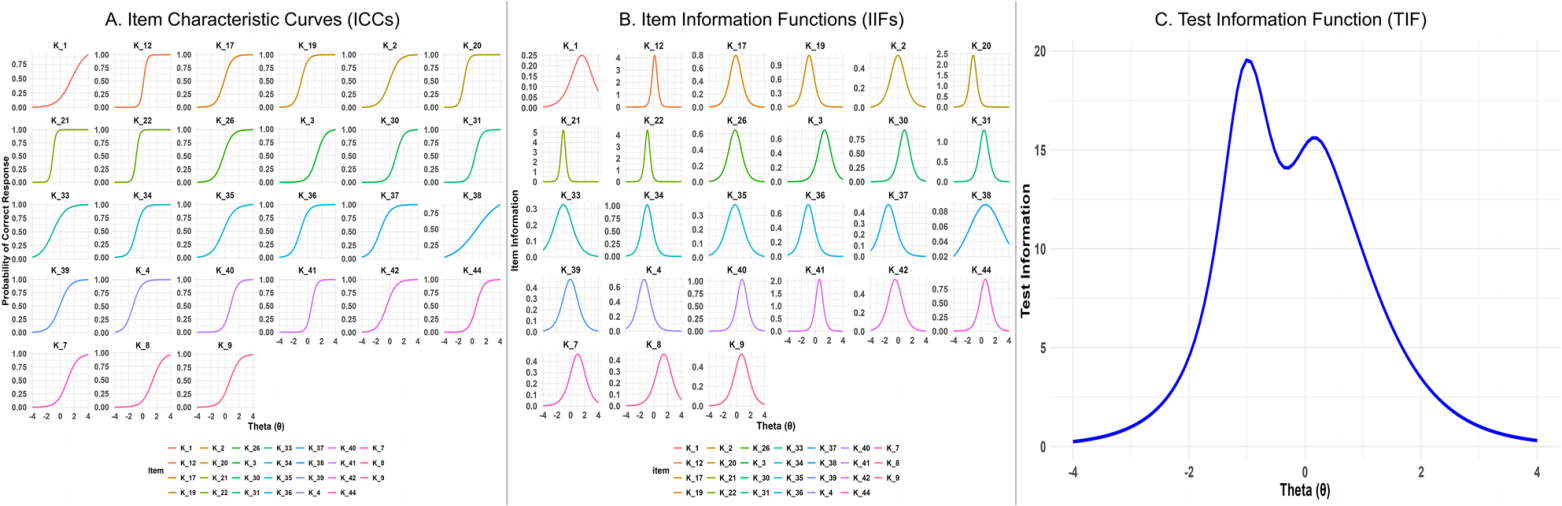
Item Response Theory (IRT) analyses for the knowledge subscale of the OH-KAP. (**A**) Item Characteristic Curves (ICCs): Each curve represents the probability of a correct response to an item across levels of the latent knowledge trait (θ). Steeper curves indicate higher item discrimination. (**B**) Item Information Functions (IIFs): These plots show how much information each item provides across the trait continuum, identifying the θ ranges where items are most precise. (**C**) Test Information Function (TIF): This curve aggregates item-level information to reflect the overall precision of the knowledge subscale. The scale performs optimally between approximately θ = − 1 and θ = 1.5, indicating the subscale is most reliable for individuals with low to moderate levels of One Health knowledge. Note: Item codes begin with “K” to indicate knowledge items

Item Characteristic Curves (ICCs) for the knowledge subscale indicate the expected monotonic increase in the probability of a correct response as a function of latent knowledge (θ), with steeper slopes reflecting greater item discrimination (Fig. 3A). Item Information Functions (IIFs) show that most items offer peak information between θ = − 0.5 and + 1.5, indicating high measurement precision in this range (Fig. 3B). This aligns with the Test Information Function (TIF), which shows maximum test-level information around θ = − 1 to + 1.5, suggesting the scale is most informative for individuals with low to moderately elevated One Health knowledge levels (Fig. 3C). Supplementary File 6 includes ICCs and IIFs for the four specific knowledge factors.

##### Cross-validation

Five-fold cross-validation results for the knowledge subscale showed consistent model fit across all data partitions. Root Mean Square Error of Approximation (RMSEA) values ranged from 0.074 to 0.081, remaining within or just below the commonly accepted threshold of 0.08.

**Table 1:** Discrimination and difficulty parameters for knowledge subscale items (*n* = 27). Items are grouped according to four specific factors identified in the final bifactor IRT model. Participants were requested to answer True, False, Don’t Know for all items

Item Code	Item Statement	Discrimination (a)	Difficulty (d)
**Factor 1: Zoonotic transmission and environmental risks**			
K_1	Close contact with livestock can lead to transmission of diseases from animals to humans.	1	-1.6
K_2	Mosquitoes and other insects can transmit diseases from animals to humans.	1.5	0.1
K_3	Animal house premises and equipment can be a source of diseases that can be transmitted from animals to humans.	1.7	-2.3
K_7	Rift Valley fever can be transmitted from animals to humans by mosquitoes.	1.4	-1.4
K_8	Rift Valley fever can be transmitted from animals to humans by handling birth products like aborted fetus and placenta.	1.3	-2
K_9	Brucellosis can be transmitted from animals to humans through drinking unboiled milk.	1.5	-1
K_30	Drinking water from communal water sources is unsafe if not boiled properly.	1.9	-1.7
K_31	Boiling milk reduces the risk of disease transmission from animals to humans.	2.3	-0.9
K_40	Floods increase the risk of food contamination with germs that can make people sick.	2	-1.6
K_41	Floods increase the risk of water contamination with germs that can make people sick.	2.9	-1.7
K_44	Uncovered meat sold in open markets can be contaminated with germs that can make people sick.	1.9	-1.1
**Factor 2: Animal bites and safe food handling**			
K_4	Animals can transmit diseases to humans by biting.	1.7	2.3
K_33	Hand washing with soap or ash before eating prevents diseases in humans.	1.1	1.2
K_34	Leaving food overnight and eating it the next day without re-cooking poses a health risk.	2	1.9
K_35	Uncooked meat may carry germs that can make people sick.	1.2	0.3
K_36	Undercooked meat may carry germs that can make people sick.	1.7	1.7
K_37	Cooking meat very well minimizes the risk of disease transmission to humans.	1.4	2
K_39	There is a human health risk if sick animals are slaughtered for human consumption.	1.4	0.1
K_42	Cholera is transmitted through food contaminated with germs.	1.5	0.7
**Factor 3: Antimicrobial resistance (AMR)**			
K_19	Not using enough of the antimicrobial dose prescribed for animals can lead germs becoming resistant.	2.1	1.9
K_20	Not using enough of the antimicrobial dose prescribed for humans can lead germs becoming resistant.	3.1	3.7
K_21	In animals, not taking antimicrobials for the prescribed number of days can lead germs to develop resistance.	4.6	4.9
K_22	In humans, not taking antimicrobials for the prescribed number of days can lead germs to develop resistance.	4.2	3.9
K_26	Antimicrobial drugs can be found in the milk of recently treated animals.	1.6	0.4
**Factor 4: Direct contact and food contamination risks**			
K_12	Anthrax can be transmitted to humans through touching the skin or hide of sick animals.	4.1	-0.5
K_17	The death of animals due to a disease can potentially result in human death from the same disease.	1.8	0.3
K_38	Mixing cooked and raw food during preparation or storage can cause food to become contaminated with germs.	0.6	-0.4

#### Attitude subscale

##### Internal consistency

The attitude subscale demonstrated excellent internal consistency, with a Cronbach’s alpha of 0.93 (95% CI: 0.92–0.95), indicating strong internal reliability and scale precision.

##### Exploratory factor analysis

**Fig. 4:**
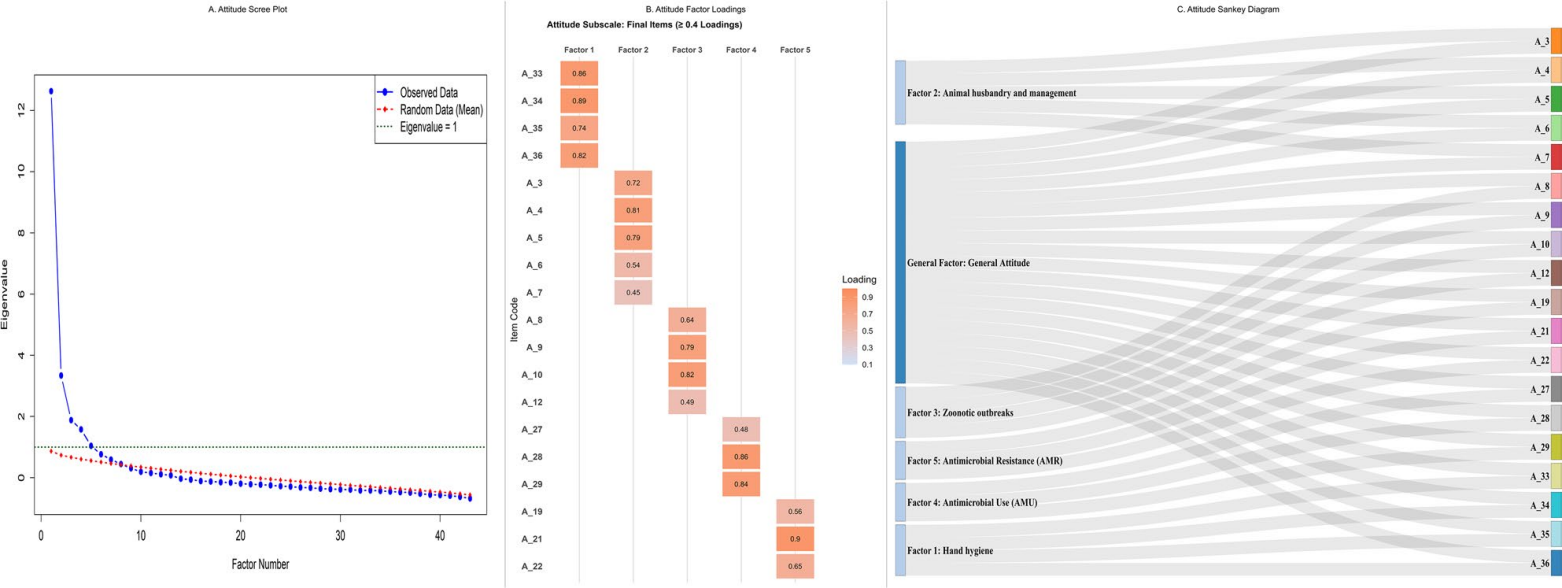
Exploratory Factor Analysis (EFA) and bifactor model results for the attitude subscale of the OH-KAP. (**A**) Parallel analysis scree plot: The solid blue line represents observed eigenvalues from the actual data; the dashed red line indicates mean eigenvalues generated from random data (parallel analysis); and the green dotted line marks the Kaiser criterion (eigenvalue = 1). Five factors were retained based on the observed eigenvalues exceeding those of the random data and visual interpretation of the scree plot. (**B**) Exploratory Factor Analysis (EFA) loadings: Standardized factor loadings (≥ 0.40) for retained items in the final model. (**C**) Sankey diagram from the bifactor IRT model: Each item loads onto the general One Health attitude factor (G) and one of five specific factors: Factor 1 – hand hygiene; Factor 2 – animal husbandry and management; Factor 3 – zoonotic outbreaks; Factor 4 – antimicrobial use (AMU); and Factor 5 – antimicrobial resistance (AMR). This structure illustrates the multidimensional yet unified nature of the One Health attitude construct. Note: Item codes begin with “A” to indicate attitude items

Bartlett’s Test of Sphericity was significant (χ² (903) = 8,091.26, *p* < 0.001), and the Kaiser–Meyer–Olkin (KMO) measure was 0.908, confirming the suitability of the data for factor analysis. Parallel analysis initially suggested seven factors; however, five were retained based on interpretability, eigenvalues > 1, and support from the scree plot (Fig. 4A), which explained 51% of the total variance. All items exhibited acceptable primary loadings (≥ 0.40), and inter-factor correlations ranged from − 0.26 to 0.44, indicating low to moderate residual associations while preserving domain specificity. These findings supported the application of a bifactor model. Factor loadings from the final EFA are presented in Fig. 4B.

#### Bifactor IRT results

##### Model structure

The bifactor graded response model for the attitude subscale comprised a general One Health attitude factor and five domain-specific factors: hand hygiene, animal husbandry and management, zoonotic outbreaks, antimicrobial use (AMU) and antimicrobial resistance (AMR) (Fig. 4C). The model included 19 polytomous items.

##### Item discrimination and thresholds

Most attitude items exhibited moderate to high discrimination (a), highlighting their capacity to distinguish individuals across varying levels of One Health-related attitudes (Table 2). High-discrimination items were spread across different content categories, including zoonoses (e.g., A_10; a = 5.3), antimicrobial use (e.g., A_28; a = 6.2), and hand hygiene (e.g., A_33; a = 3.1). Only one item (A_5; a = 1.1) approached the lower end of the acceptable range, indicating that nearly all items contributed meaningfully to the measurement model. Threshold estimates (d₁, d₂) showed substantial variability, capturing a broad spectrum of endorsement difficulty. Items with low thresholds (e.g., A_4; d₂ = − 3.3) were likely to be endorsed even by respondents with less favorable attitudes, whereas items such as A_21 (d₁ = 9.2, d₂ = 0.5) required stronger agreement with positive One Health attitudes, marking them as useful indicators of more advanced attitudinal positioning.

##### Model fit and local dependence

The bifactor graded response model demonstrated excellent overall fit to the attitude data: M²(114) = 187.82, *p* < 0.001; RMSEA = 0.047 (90% CI: 0.034–0.058); SRMSR = 0.076; CFI = 0.988; TLI = 0.984. Most items exhibited satisfactory local fit, as indicated by residual correlation patterns and local dependence diagnostics (Supplementary File 7). A small number of items (e.g., A_3, A_29) showed signs of potential local dependence, likely stemming from conceptual or linguistic overlap. These items were retained based on their substantive importance to the domain content. Taken together, the fit indices and local diagnostics support the structural soundness of the model and affirm the validity of the general attitude factor.

##### Item and test information

**Fig. 5:**
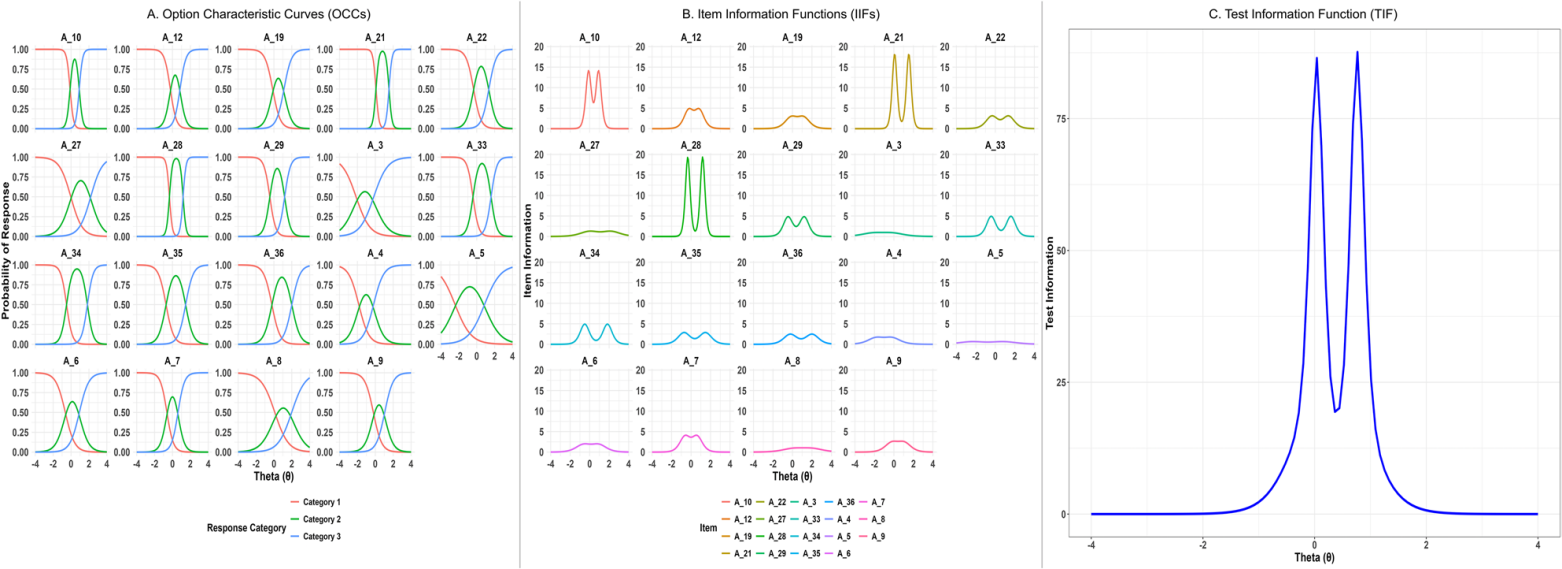
Item Response Theory (IRT) analyses for the attitude subscale of the OH-KAP. (**A**) Option Characteristic Curves (OCCs): These curves depict the probability of selecting each response category (1 = red, 2 = green, 3 = blue) as a function of θ. Category curves crossing at distinct points demonstrate meaningful response differentiation. (**B**) Item Information Functions (IIFs): These plots show the amount of information each attitude item provides across the latent trait continuum (θ). Peaks indicate the trait levels where each item offers the greatest measurement precision. (**C**) Test Information Function (TIF): This curve aggregates the information provided by all items, indicating how reliably the attitude subscale measures the underlying trait. The test provides the highest precision around θ = 0, reflecting optimal measurement for respondents with moderate levels of One Health attitude

Option Characteristic Curves (OCCs) indicated well-functioning polytomous items, with ordered and distinct response category curves across the latent attitude continuum (θ) (Fig. 5A). Item Information Functions (IIFs) showed that several items provided strong measurement precision between θ = 0 and + 1.5, including A_33, A_28, and A_21 (Fig. 5B). The Test Information Function (TIF) peaked sharply around θ = 0, with twin peaks suggesting optimal reliability for individuals with average to moderately high levels of One Health attitudes (Fig. 5C). Measurement precision declined outside this range, consistent with a scale targeted toward the mid-spectrum of the latent trait. Supplementary File 8 includes the OCCs and IIFs for the five domain-specific attitude factors.

##### Cross-validation

Five-fold cross-validation results for the knowledge subscale showed consistent model fit across all data partitions. Root Mean Square Error of Approximation (RMSEA) values ranged from 0.074 to 0.081, remaining within or just below the commonly accepted threshold of 0.08, confirming the generalizability and structural stability of the bifactor model.

**Table 2:** Discrimination and difficulty thresholds for attitude subscale items (*n* = 19). Items are grouped according to five specific factors identified in the final bifactor IRT model. Superscripts on item codes indicate the response scale used: ^1^Very Important / Somewhat Important / Not Important / Don’t know; ^2^Strongly Agree / Somewhat Agree / Don’t Agree / Don’t know; ^3^Very Concerned / Somewhat Concerned / Not Concerned / Don’t know; ^4^Very Risky / Somewhat Risky / Not Risky / Don’t know

Item Code	Item Statement	Discrimination (a)	Difficulty Threshold 1 (d1)	Difficulty Threshold 2 (d2)
**Factor 1: Hand hygiene**				
A_33^1^	How important do you think it is to wash your hands with soap after using a latrine or practicing open defecation?	3.1	5.0	-1.3
A_34^1^	How important is it for your health to wash hands before handling food?	3.1	5.7	-1.6
A_35^1^	How important is it for your health to wash hands after handling food?	2.4	3.5	-1.7
A_36^2^	Using soap and water kills germs much more effectively for hand cleaning compared to using only water.	2.2	4.5	-0.5
**Factor 2: Animal husbandry and management**				
A_3^4^	How risky is it for children’s health to play with animal manure?	1.3	-0.2	-2.8
A_4^3^	How concerned are you about contracting diseases when clearing animal manure?	1.8	-0.4	-3.3
A_5^1^	How important is it to wear protective clothing when slaughtering animals?	1.1	1.0	-2.7
A_6^3^	How concerned are you about getting diseases from your animals if they get mixed with other herds?	1.9	1.8	-1.2
A_7^3^	How concerned are you about getting diseases from your animals if your livestock cross international borders?	2.8	1.7	-1.7
**Factor 3: Zoonotic outbreaks**				
A_8^3^	How concerned are you about getting a disease if an animal bites you?	1.3	2.6	0.1
A_9^2^	When mosquitoes are abundant during flooding, I am afraid of catching Rift Valley Fever disease.	2.2	2.3	-0.5
A_10^2^	When many animals are dying in my area, I am afraid of contracting a disease if I eat the meat of such animals.	5.3	4.9	-0.6
A_12^3^	How concerned are you about diseases spreading to humans during an outbreak of animal disease?	3.0	2.5	-0.7
**Factor 4: Antimicrobial use (AMU)**				
A_27^2^	The more antimicrobial dose I give to the animal, the higher the chance that the animal will recover.	1.6	3.5	0.0
A_28^2^	When animals show improvement, there is no need to complete the entire prescribed course.	6.2	7.4	-2.1
A_29^2^	When humans show improvement, there is no need to complete the entire prescribed course.	3.1	3.7	-1.4
**Factor 5: Antimicrobial resistance (AMR)**				
A_19^3^	To what extent are you concerned that germs could become resistant when antimicrobials are used to treat humans?	2.4	2.7	-0.3
A_21^3^	How concerned are you about germs developing resistance when using antimicrobial drugs for yourself/family without a prescription?	6.0	9.2	0.5
A_22^3^	How concerned are you about germs developing resistance when using antimicrobial drugs for your animals without a prescription?	2.5	3.4	-0.9

#### Practice subscale

**Fig. 6:**
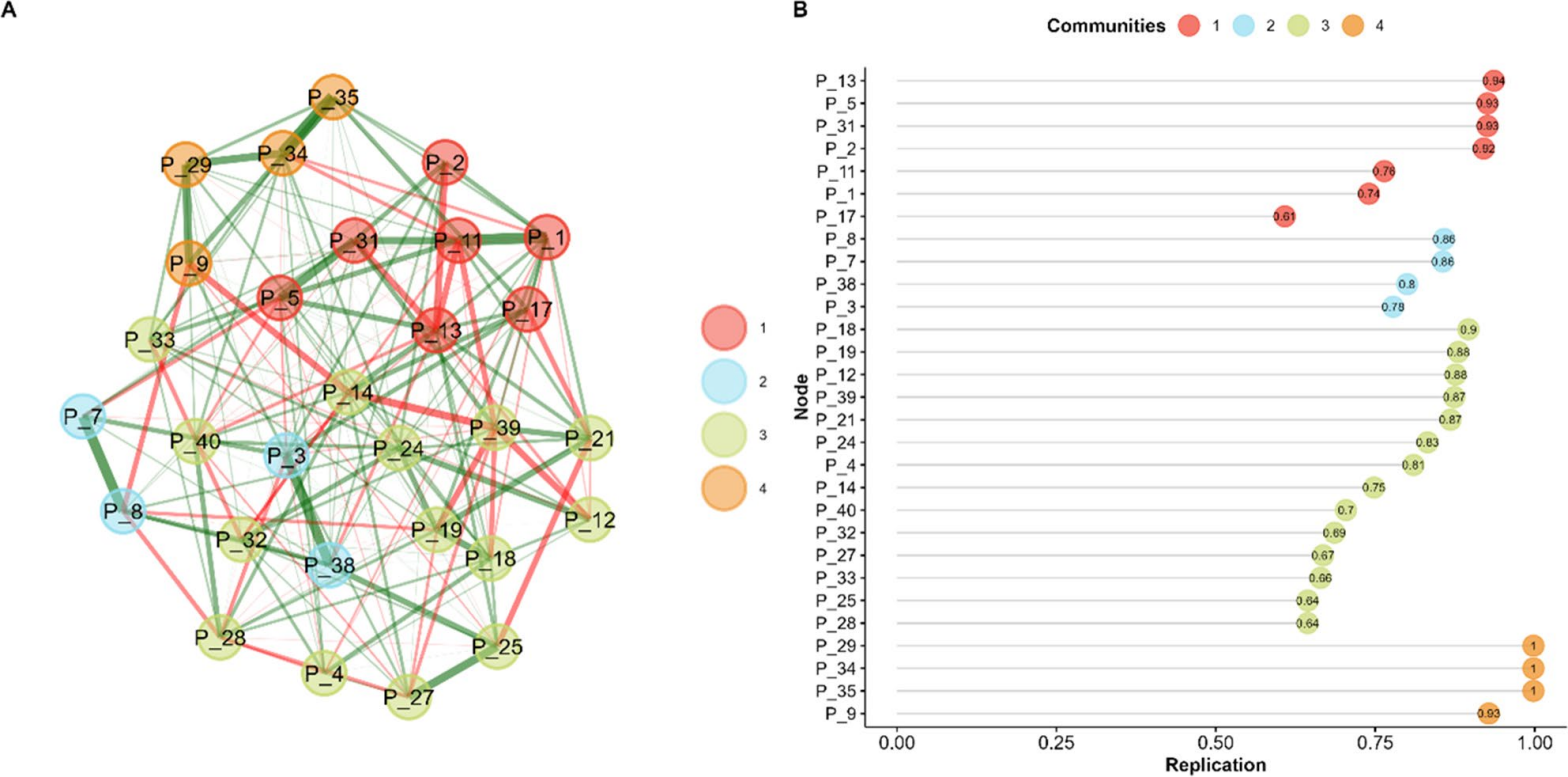
Exploratory Graph Analysis (EGA) results for the practice subscale. (**A**) Network plot of the practice subscale, where each circle (node) represents an item and lines (edges) represent associations between items. Node colours indicate the four identified practice communities: (1) animal management and antimicrobial use, (2) direct contact & exposure control, (3) responsible husbandry, food safety, and antimicrobial stewardship, and (4) hand hygiene & disease reporting. Thicker lines indicate stronger associations; green lines are positive and red lines are negative. (**B**) Item stability plot showing the proportion of bootstrap samples (*n* = 500) in which each item replicated in its assigned community. Higher values indicate greater stability, with most items demonstrating strong replication across bootstrap samples

Analysis of the practice subscale revealed a clear, multi-community structure after removing redundant and unstable items. Redundancy analysis identified eight highly overlapping items, which were removed before modelling. Initial EGA of the reduced dataset suggested a four-community structure as the most frequent solution in bootstrap resampling (51.8% of 500 iterations) (Fig. 6A). Item stability analysis revealed two items with low replication (< 50% consistency), which were removed. Repeating EGA on the cleaned dataset again supported a four-community structure, now with greater stability (66.8% of bootstrap samples; 95% CI: 2.68–5.32 communities). Item stability for the final model was high, with most items replicating in their assigned community in the majority of bootstrap samples (Fig. 6B). The final model identified four item communities. Standardized network loadings from the EGA model were calculated to quantify the strength of association between each item and the extracted dimensions representing the underlying constructs (Table 3).

**Table 3:** Standardized network loadings for the practice subscale, arranged by the four communities identified in the final Exploratory Graph Analysis (EGA). Items are grouped under their respective communities. The four columns show standardized network loadings on Dimensions 1–4 from the EGA model; these dimensions are not identical to the communities but represent the underlying factors extracted by the model, with higher absolute values indicating stronger item–dimension associations. Participants responded using the following options: Yes, always; Yes, sometimes; No, never; Don’t know/Don’t want to answer

Item Code	Item Statement	Dimension 1 (network loadings)	Dimension 2 (network loadings)	Dimension 3 (network loadings)	Dimension 4 (network loadings)
**Community 1: Animal Management and Antimicrobial Use**					
P_1	Do you and your livestock sleep under the same roof?	0.22	0.00	-0.14	-0.03
P_2	Do you and your livestock share the same drinking water?	0.39	0.17	0.07	0.05
P_5	Do you dispose dead animals by burying, burning or combination of both?	0.20	-0.15	0.11	-0.02
P_11	If you were bitten by a dog, would you seek healthcare?	0.73	0.00	-0.05	0.00
P_13	Do you reuse leftover antimicrobial drugs after the bottle has been opened for some time?	-0.34	-0.12	0.01	-0.01
P_17	Do you use antimicrobials to fatten your livestock for better production?	-0.03	0.10	0.24	0.09
P_31	Do you consume undercooked liver?	0.68	0.14	-0.12	0.03
**Community 2: Direct contact & exposure control**					
P_3	Do you slaughter sick animals for human consumption before they die?	-0.01	0.43	-0.02	0.08
P_7	Do you ever touch animal manure with your bare hands?	0.00	0.49	0.01	0.00
P_8	Do you ever touch birth products with your bare hands?	0.02	0.40	-0.01	-0.10
P_38	Do you slaughter livestock inside your residential dwelling?	0.15	0.43	0.24	0.03
**Community 3: Responsible Husbandry**,** Food Safety**,** and Antimicrobial Stewardship**					
P_4	Do you vaccinate your livestock against diseases that can be transmitted from animals to humans?	0.03	-0.03	0.18	0.06
P_12	Do you isolate your animals when they are sick?	0.02	0.00	0.43	0.00
P_14	Do you ever give expired antimicrobials to animals?	-0.18	0.00	0.44	-0.10
P_18	Do you seek advice from an animal health professional before giving antimicrobials to your livestock?	-0.09	0.00	0.61	0.08
P_19	Do you consult with a human health professional before taking antimicrobials yourself or giving them to a family member?	0.08	-0.08	0.37	0.00
P_21	Do you follow the recommended course of antimicrobial treatment in animals?	0.18	-0.01	-0.07	0.01
P_24	Do you abstain from eating meat of recently treated animals?	0.07	0.09	0.47	0.08
P_25	Do you increase the antimicrobial dose if the animal does not recover after the completion of the treatment?	-0.07	0.14	0.42	-0.03
P_27	Do you change the antimicrobial drug without consulting an animal health professional if the initial treatment does not work?	-0.02	0.06	0.21	0.01
P_28	Do you cover food to prevent flies from touching the food?	-0.01	-0.21	0.16	0.00
P_32	Do you drink unboiled milk?	-0.06	0.20	0.03	0.03
P_33	Do you clean animal udder before milking?	0.13	0.00	0.08	0.15
P_39	Do you eat meat from wild animals?	0.07	-0.05	-0.68	0.00
P_40	If you prepare food but don’t eat it right away, do you reheat it thoroughly before consuming?	-0.05	0.02	0.23	0.09
**Community 4: Hand hygiene & disease reporting**					
P_9	Do you report animal disease outbreaks to community animal health workers or veterinary authorities?	0.00	-0.05	-0.18	0.36
P_29	Do you use soap or ash to wash your hands before handling food?	0.08	0.02	0.19	0.51
P_34	Do you wash your hands with soap or ash after using a latrine or practicing open defecation?	-0.16	0.01	0.27	0.54
P_35	Do you wash your hands with water and soap/ash before eating?	0.04	0.03	0.08	0.57

## Discussion

This study is the first, to our knowledge, to develop and rigorously validate a unified KAP instrument spanning topics typically addressed by One Health initiatives, namely: zoonoses, AMR, and food safety hazards. By combining content validation with advanced analytical approaches such as IRT and EGA, the OH-KAP overcomes long-standing methodological limitations in One Health KAP research. Although the instrument was designed and validated for use in (agro-)pastoralist and mixed-farming settings in the Horn of Africa, the developed methodology and the tool itself will likely have broader application.

Most previous One Health–related KAP surveys in rural settings have relied on classical test theory, reporting mainly internal consistency but rarely exploring deeper construct validity or item-level diagnostics [3, 42, 44, 50]. Some surveys have focused primarily on whether communities have heard of ‘One Health,’ without defining what it means to know One Health as an integrated construct [33]. This means respondents may demonstrate relevant knowledge, attitudes, and practices despite never having encountered the term itself. Some studies have used advanced psychometrics models such as 2PL IRT for livestock-associated zoonoses in Ethiopia [4, 5], although these have generally focused on single-domain instruments rather than capturing the broader integrated One Health construct. The present study marks a step forward by conceiving One Health as a single integrated construct across zoonoses, AMR, and food safety. It applies bifactor IRT models to capture both general and domain-specific variance, using network psychometrics to structure diverse practices into stable communities; and implementing five-fold cross-validation to confirm model stability, an approach rarely reported in KAP literature. These methodological innovations directly support the standardization and reproducibility of the survey, ensuring its findings are reliable and comparable across different contexts.

Development and validation of the OH-KAP followed two phases, including content validation by experts and psychometric evaluation of responses following field testing. Content validation demonstrated strong expert agreement on items that were relevant to the construct of One Health (S-CVI/Ave = 0.903). In psychometric evaluation, EFA supported four interpretable factors for knowledge, and a bifactor 2PL IRT model provided superior fit, with measurement precision highest for low-to-moderately elevated knowledge levels. For attitudes, EFA supported five factors, and a bifactor graded response model (GRM) achieved excellent fit, with optimal precision at average-to-moderately high attitude levels. The bifactor IRT solutions for both subscales confirmed a hierarchical structure: a strong general One Health factor complemented by domain-specific dimensions. This supports the use of both overall scores (for broad assessment) and domain-level scores (for targeted intervention planning). Practices, typically more multifaceted, were best represented through a stable four-community structure identified using exploratory graph analysis (EGA) after removing redundant items to improve clarity. This approach addressed the common problem of low internal consistency for diverse behaviours and provided a clearer, more reliable representation of practice patterns, allowing for nuanced assessment rather than treating practices as a single undifferentiated construct.

Whether used as a cross-sectional survey or before and after an intervention, the OH-KAP is suitable for exploring how communities perceive complex, interconnected health risks. It helps uncover nuanced understandings and misperceptions that may underlie behaviours, supporting the design of more context-specific and multi-sectoral interventions. The OH-KAP can be administered to adults (≥ 18 years) by trained enumerators in the local language, with thumbprint consent acceptable for participants with limited literacy. Scoring should apply fixed parameters from the original validation (reported in this study) to maintain comparability. Supplementary File 9 provides implementing and scoring instructions. General factor scores offer a rapid snapshot of a community’s overall preparedness to adopt and sustain One Health behaviours, while domain-level scores and practice communities identify specific behavioural or knowledge gaps. This dual-level reporting allows One Health practitioners to align resources more efficiently, design domain-specific education strategies, and integrate findings into One Health surveillance systems, amongst other areas. For example, a population scoring low in the ‘direct contact and exposure control’ practice community could receive targeted training on manure handling, slaughter hygiene, and locally appropriate protective measures, whereas high AMR knowledge but low AMR-related attitudes might prompt a focus on attitude-shifting campaigns.

Beyond its application in programme design, the tool is well-suited for enumerator-led administration, making it accessible for low-literacy populations and reducing barriers to data collection in remote settings. Its psychometric strength means that changes observed over time are more likely to represent real differences rather than random variation, supporting reliable monitoring of integrated One Health initiatives. The tool’s design and scope align with the WHO Joint Plan of Action (2022–2026) [48] and the Lancet One Health Commission [60], supporting the delivery of their recommendations on community engagement in One Health and providing governments and NGOs with a robust, scalable instrument for tracking change in vulnerable communities.

This study has notable strengths, including a systematic two-phase development process, a diverse expert panel for content validation, the use of modern psychometric methods tailored to item type, and contextual adaptation for pastoral settings. Nevertheless, certain limitations should be acknowledged. A small subset of items showed local dependence, meaning that responses to certain items were more closely related to each other than could be explained by the underlying construct alone; these were retained to ensure important content areas were covered. Measurement precision was lower at the extreme high and low ends of the scale and indicates reduced accuracy for respondents with very high or very low levels of the trait. The participant-to-item ratio of 2.4 is lower than conventional recommendations; however, stable factor structures with strong loadings and excellent model fit support the adequacy of our sample [22]. The validation sample was drawn exclusively from one administrative region of Somalia. While the tool has also been adapted and applied in Ethiopia and Kenya [43], generalizability across the full diversity of African pastoral and rural communities cannot be assumed without further formal external validation across additional geographic, linguistic, and livelihood contexts. Logistical constraints in remote pastoralist and agropastoralist communities further limited the feasibility of larger samples. Finally, while this study advances One Health measurement by modelling zoonoses, AMR, and food safety as integrated constructs, these domains do not capture the full scope of One Health. Important dimensions such as ecosystem health, climate change, and socio-economic drivers were beyond the scope of the present scale and warrant consideration in future instrument development.

Because the study was conducted in the Somali context, cultural–linguistic adaptation and external validation will be essential for broader application. Future research should therefore assess the instrument’s applicability across diverse geographic, cultural, and livelihood settings. Key priorities include testing measurement invariance across literacy levels, gender, and age groups to ensure comparability; conducting cross-cultural validation in communities beyond the Horn of Africa; undertaking longitudinal responsiveness studies to assess the tool’s sensitivity to change in response to interventions; and developing short-form adaptations for rapid deployment in emergency or large-scale surveys. Addressing these areas will strengthen the tool’s utility for diverse One Health initiatives and support the establishment of validated norms and benchmarks across regions.

## Conclusion

This study developed and validated the One Health Knowledge, Attitudes and Practices (OH-KAP) instrument, a concise and psychometrically robust tool for assessing how rural communities understand and act on interconnected risks across human, animal, and environmental health. By combining bifactor item response theory for knowledge and attitudes with network psychometrics for practice items, the OH-KAP advances measurement beyond traditional KAP surveys, offering both a general One Health construct and domain-specific insights. The tool can support baseline assessments, programme evaluation, and the design of targeted interventions in rural settings. Future work should extend coverage to additional One Health dimensions, evaluate cross-cultural applicability, and test longitudinal sensitivity to behaviour change.

## Supplementary Information

Below is the link to the electronic supplementary material.

Supplementary Material 1

Supplementary Material 2

Supplementary Material 3

Supplementary Material 4

Supplementary Material 5

Supplementary Material 6

Supplementary Material 7

Supplementary Material 8

Supplementary Material 9

## Data Availability

The raw field test data used for this study will be deposited in the University of Liverpool’s Data CatLog repository. The corresponding access link will be included here prior to the publication of the study.

## Abbreviations

AIC: Akaike Information Criterion
AMR: Antimicrobial Resistance
AMU: Antimicrobial Use
BIC: Bayesian Information Criterion
CFI: Comparative Fit Index
CI: Confidence Interval
CVI: Content Validity Index
EFA: Exploratory Factor Analysis
EGA: Exploratory Graph Analysis
GRM: Graded Response Model
ICC: Item Characteristic Curve
IIF: Item Information Function
IRT: Item Response Theory
KAP: Knowledge, Attitude, Practice
KMO: Kaiser–Meyer–Olkin measure
MINRES: Minimum Residual
OH: One Health
RMSEA: Root Mean Square Error of Approximation
SRMSR: Standardized Root Mean Square Residual
TIF: Test Information Function
TLI: Tucker-Lewis Index
WHO: World Health Organization
WOAH: World Organisation for Animal Health
