# Supplementary material for: Development and validation of the OH-KAP survey for use with pastoral and other rural communities in Africa
Source: One Health Outlook. 2026 May 22;8:36. doi: 10.1186/s42522-026-00213-8 (PMC13404092; doi:10.1186/s42522-026-00213-8)
Supplement: Supplementary file 5 — Supplementary Material 5 [file 42522_2026_213_MOESM5_ESM.docx]

Supplementary File 6. Item-level local fit statistics for the Knowledge subscale under the bifactor IRT model. Columns show the S-X² item fit statistic (S_X2), its associated degrees of freedom (df.S_X2), RMSEA derived from the S-X² test (RMSEA.S_X2), and the corresponding p-value (p.S_X2). Items with low p-values (e.g., K_35, K_38) indicate potential local dependence or misfit, though these were retained for their conceptual relevance and contribution to content validity. Participants were requested to answer True, False, Don’t Know for all items.

| **Item Code** | **Item statement** | **S_X2** | **df.S_X2** | **RMSEA.S_X2** | **p.S_X2** |
| --- | --- | --- | --- | --- | --- |
| K_1 | Close contact with livestock can lead to transmission of diseases from animals to humans. | 31.11 | 17.00 | 0.05 | 0.02 |
| K_2 | Mosquitoes and other insects can transmit diseases from animals to humans. | 25.50 | 15.00 | 0.05 | 0.04 |
| K_3 | Animal house premises and equipment can be a source of diseases that can be transmitted from animals to humans. | 10.88 | 12.00 | 0.00 | 0.54 |
| K_4 | Animals can transmit diseases to humans by biting. | 19.80 | 12.00 | 0.05 | 0.07 |
| K_7 | Rift Valley fever can be transmitted from animals to humans by mosquitoes. | 20.79 | 16.00 | 0.03 | 0.19 |
| K_8 | Rift Valley fever can be transmitted from animals to humans by handling birth products like aborted fetus and placenta. | 18.47 | 14.00 | 0.03 | 0.19 |
| K_9 | Brucellosis can be transmitted from animals to humans through drinking unboiled milk. | 32.48 | 17.00 | 0.06 | 0.01 |
| K_12 | Anthrax can be transmitted to humans through touching the skin or hide of sick animals. | 42.26 | 20.00 | 0.06 | 0.00 |
| K_17 | The death of animals due to a disease can potentially result in human death from the same disease. | 31.32 | 17.00 | 0.05 | 0.02 |
| K_19 | Not using enough of the antimicrobial dose prescribed for animals can lead germs becoming resistant. | 35.65 | 14.00 | 0.07 | 0.00 |
| K_20 | Not using enough of the antimicrobial dose prescribed for humans can lead germs becoming resistant. | 28.72 | 12.00 | 0.07 | 0.00 |
| K_21 | In animals, not taking antimicrobials for the prescribed number of days can lead germs to develop resistance. | 39.88 | 9.00 | 0.11 | 0.00 |
| K_22 | In humans, not taking antimicrobials for the prescribed number of days can lead germs to develop resistance. | 27.76 | 9.00 | 0.08 | 0.00 |
| K_26 | Antimicrobial drugs can be found in the milk of recently treated animals. | 19.73 | 15.00 | 0.03 | 0.18 |
| K_30 | Drinking water from communal water sources is unsafe if not boiled properly. | 27.88 | 14.00 | 0.06 | 0.01 |
| K_31 | Boiling milk reduces the risk of disease transmission from animals to humans. | 8.55 | 12.00 | 0.00 | 0.74 |
| K_33 | Hand washing with soap or ash before eating prevents diseases in humans. | 35.99 | 15.00 | 0.07 | 0.00 |
| K_34 | Leaving food overnight and eating it the next day without re-cooking poses a health risk. | 14.78 | 10.00 | 0.04 | 0.14 |
| K_35 | Uncooked meat may carry germs that can make people sick. | 36.56 | 19.00 | 0.06 | 0.01 |
| K_36 | Undercooked meat may carry germs that can make people sick. | 24.60 | 16.00 | 0.04 | 0.08 |
| K_37 | Cooking meat very well minimizes the risk of disease transmission to humans. | 29.14 | 16.00 | 0.05 | 0.02 |
| K_38 | Mixing cooked and raw food during preparation or storage can cause food to become contaminated with germs. | 49.45 | 23.00 | 0.06 | 0.00 |
| K_39 | There is a human health risk if sick animals are slaughtered for human consumption. | 12.55 | 14.00 | 0.00 | 0.56 |
| K_40 | Floods increase the risk of food contamination with germs that can make people sick. | 20.51 | 20.00 | 0.01 | 0.43 |
| K_41 | Floods increase the risk of water contamination with germs that can make people sick. | 23.38 | 18.00 | 0.03 | 0.18 |
| K_42 | Cholera is transmitted through food contaminated with germs. | 10.41 | 13.00 | 0.00 | 0.66 |
| K_44 | Uncovered meat sold in open markets can be contaminated with germs that can make people sick. | 14.45 | 12.00 | 0.03 | 0.27 |
