# Supplementary material for: Development and validation of the OH-KAP survey for use with pastoral and other rural communities in Africa
Source: One Health Outlook. 2026 May 22;8:36. doi: 10.1186/s42522-026-00213-8 (PMC13404092; doi:10.1186/s42522-026-00213-8)

***Supplementary File 6.*** *This file provides the item characteristic curves (ICCs) and item information functions (IIFs) for the specific four factors (S1-S4) of the Knowledge subscale from the bifactor IRT model. In the main manuscript, we present only the general One Health factor plots and the test information function (TIF). The domain-specific plots are included here for completeness.*


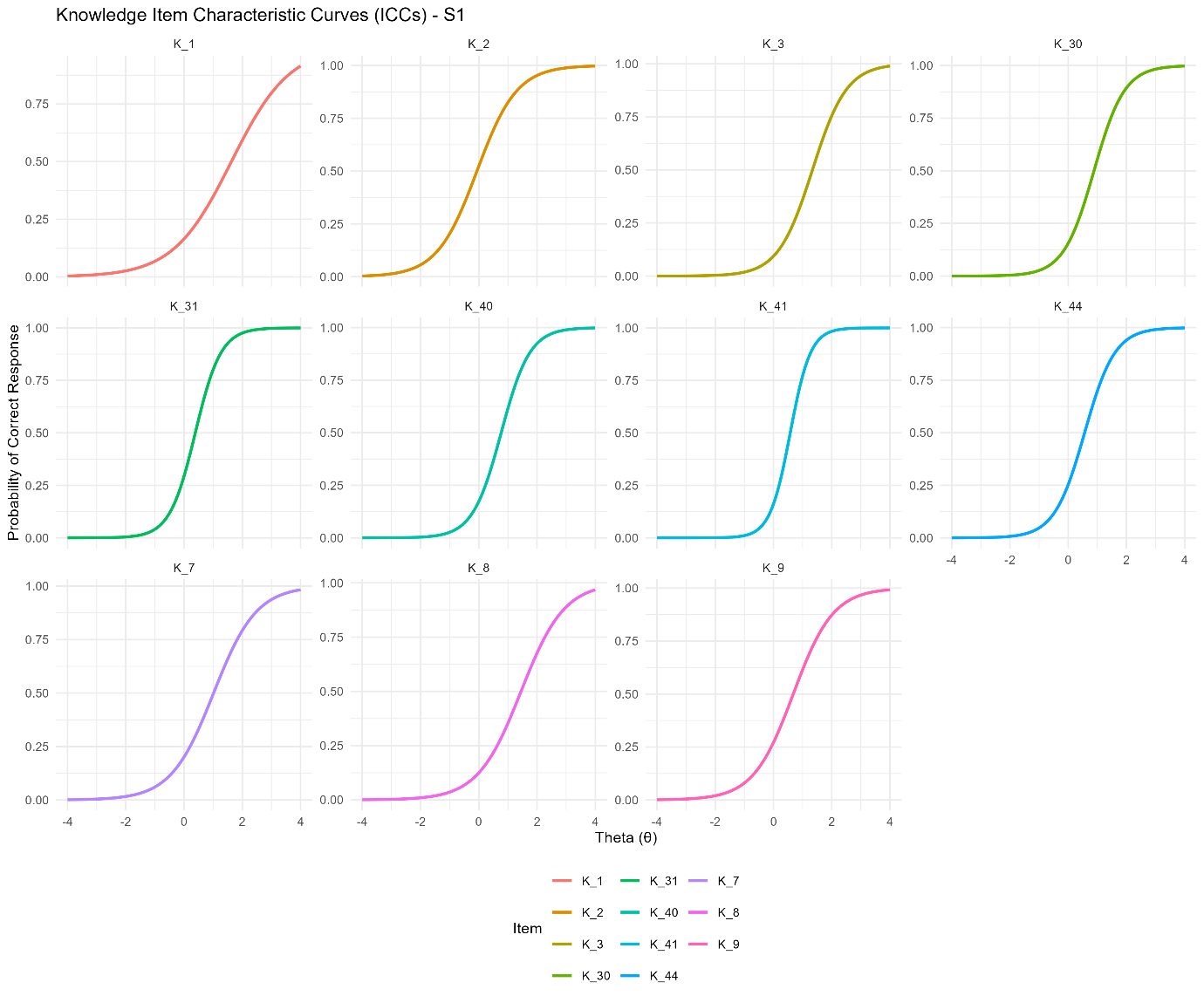


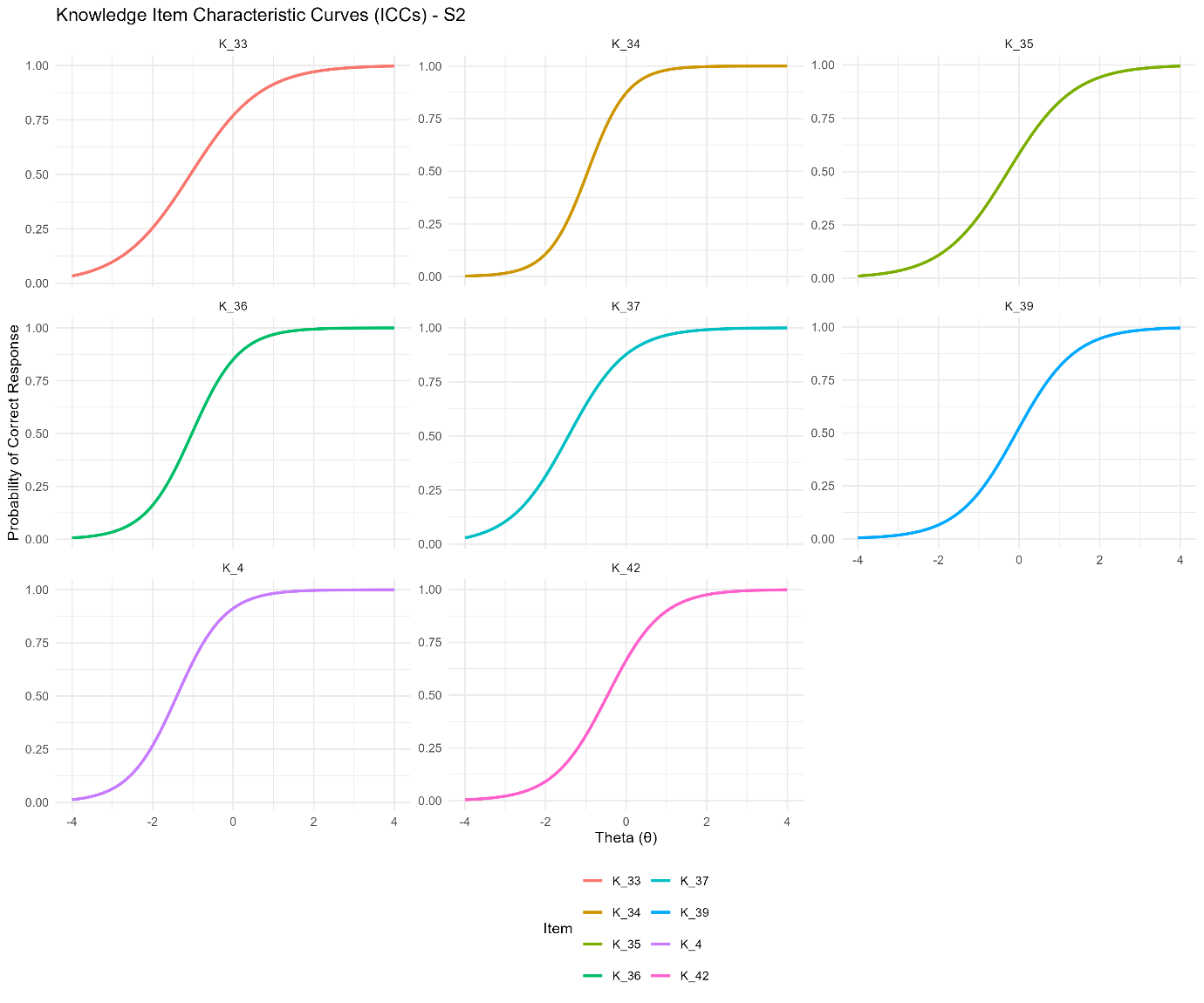

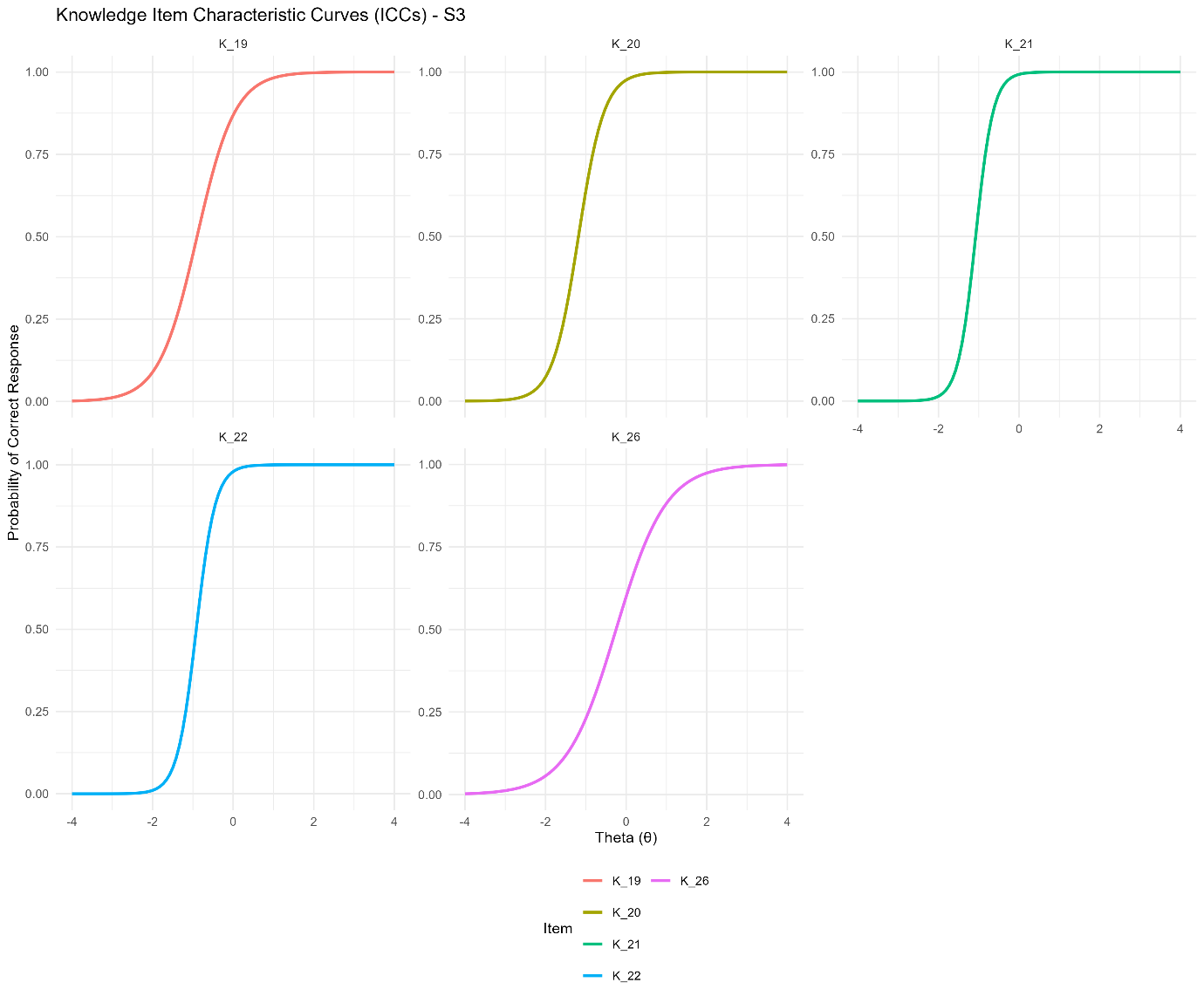

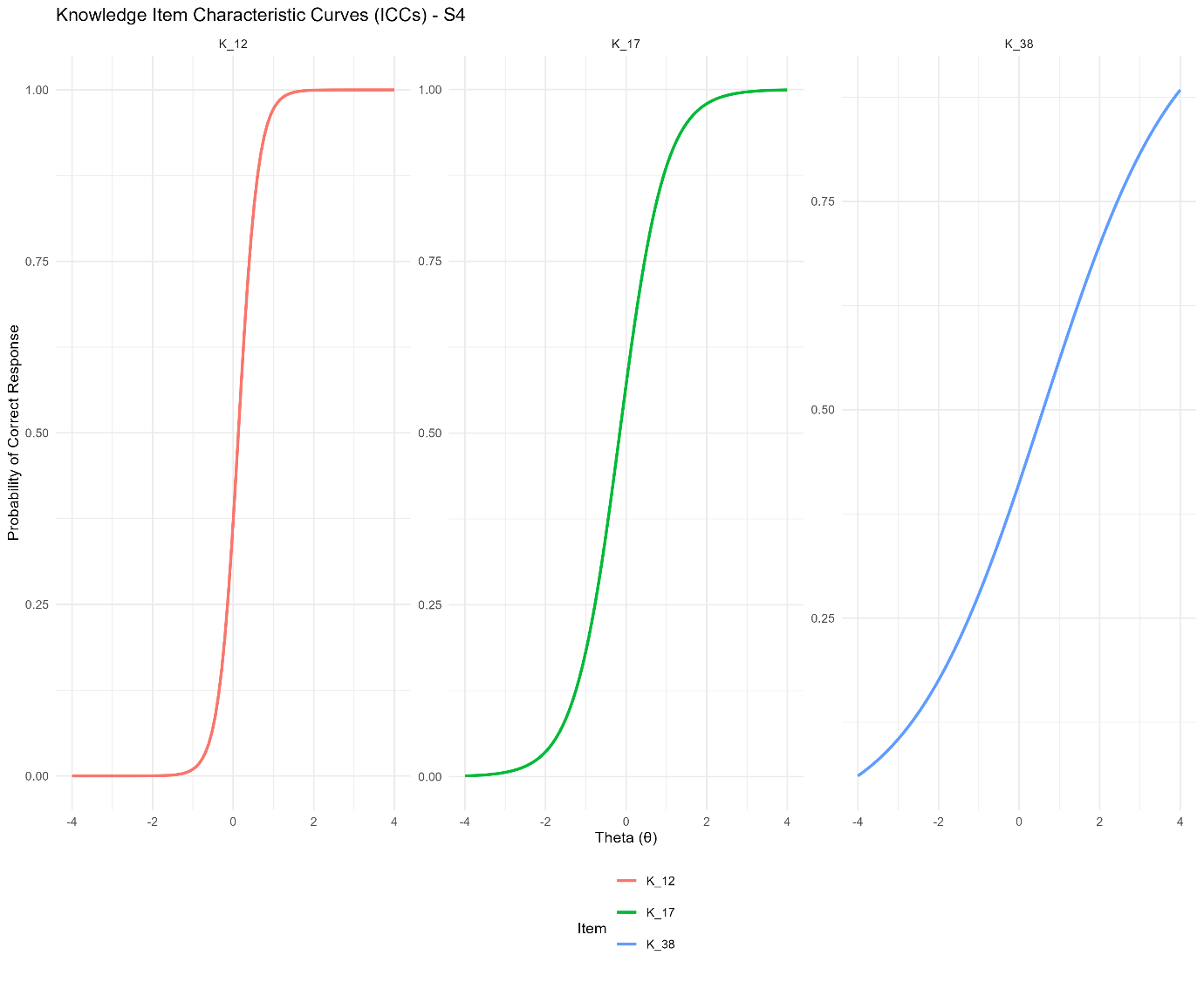

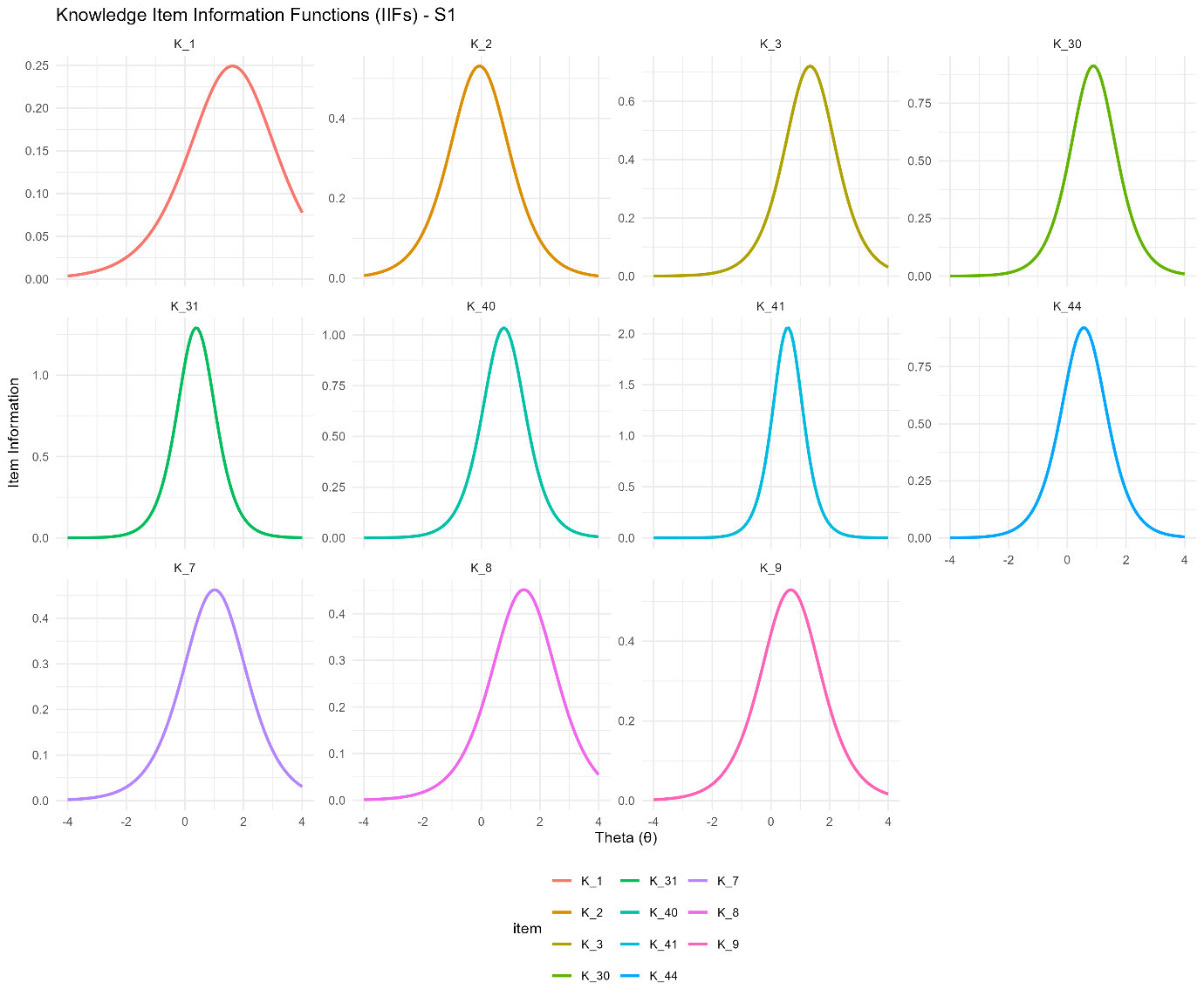

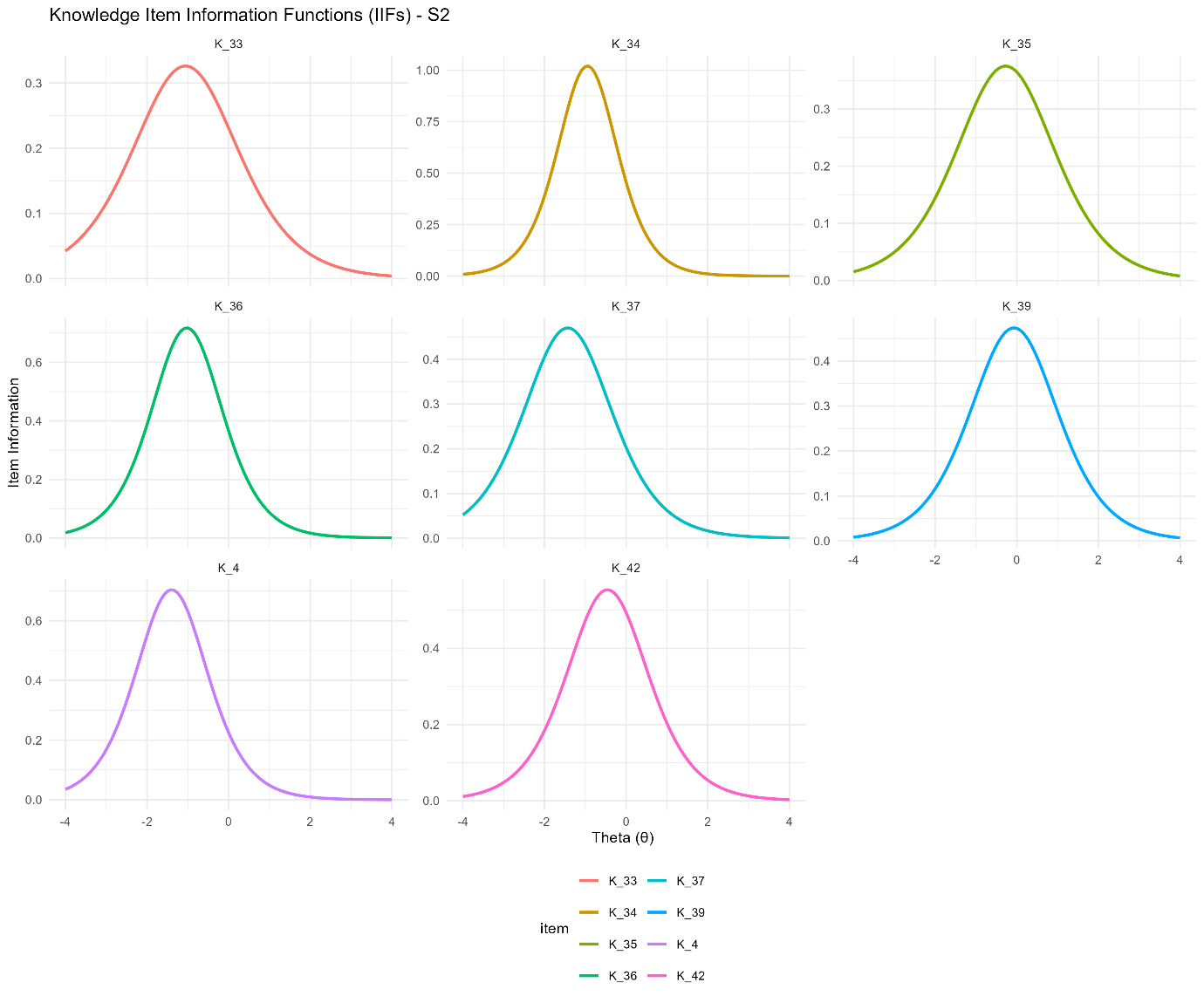

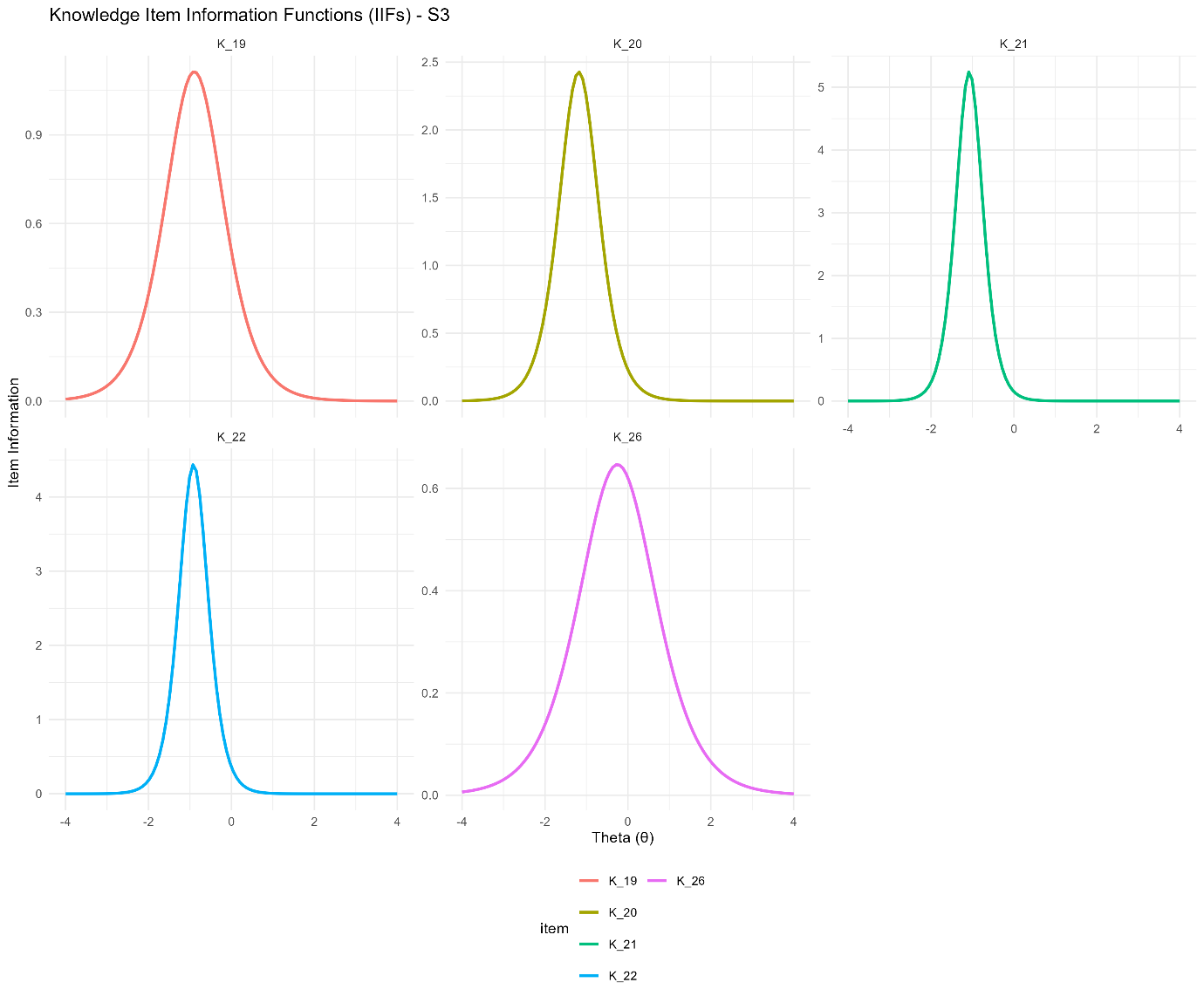

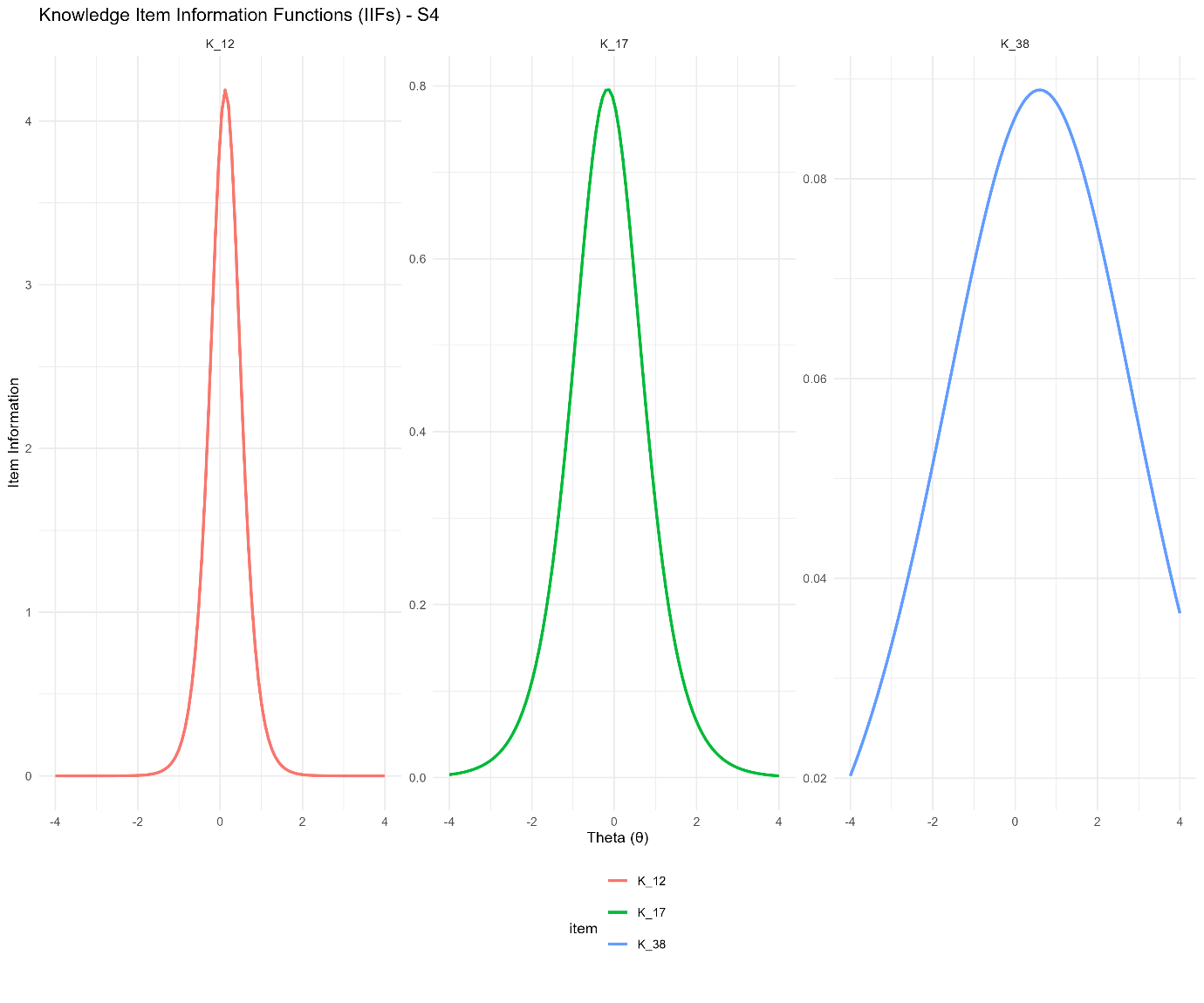

Supplement: Supplementary file 6 — Supplementary Material 6 [file 42522_2026_213_MOESM6_ESM.docx]
