# Supplementary material for: Development and validation of the OH-KAP survey for use with pastoral and other rural communities in Africa
Source: One Health Outlook. 2026 May 22;8:36. doi: 10.1186/s42522-026-00213-8 (PMC13404092; doi:10.1186/s42522-026-00213-8)
Supplement: Supplementary file 7 — Supplementary Material 7 [file 42522_2026_213_MOESM7_ESM.docx]

Supplementary File 8. Item-level local fit statistics for the Attitude subscale under the bifactor graded response model. Columns present each item code and statement, the S-X² item fit statistic (S_X2), associated degrees of freedom (df.S_X2), RMSEA derived from the S-X² test (RMSEA.S_X2), and the corresponding p-value (p.S_X2). Items with low p-values (e.g., A_3, A_29) suggest potential local dependence; however, they were retained based on conceptual and content relevance. Superscripts on item codes indicate the response scale used: 1Very Important / Somewhat Important / Not Important / Don’t know; 2Strongly Agree / Somewhat Agree / Don’t Agree / Don’t know; 3Very Concerned / Somewhat Concerned / Not Concerned / Don’t know; 4Very Risky / Somewhat Risky / Not Risky / Don’t know.

| **Item Code** | **Item statement** | **S_X2** | **df.S_X2** | **RMSEA.S_X2** | **p.S_X2** |
| --- | --- | --- | --- | --- | --- |
| A_3^4^ | How risky is it for children's health to play with animal manure? | 58.78 | 28.00 | 0.06 | 0.00 |
| A_4^3^ | How concerned are you about contracting diseases when clearing animal manure? | 33.92 | 18.00 | 0.05 | 0.01 |
| A_5^1^ | How important is it to wear protective clothing when slaughtering animals? | 67.35 | 31.00 | 0.06 | 0.00 |
| A_6^3^ | How concerned are you about getting diseases from your animals if they get mixed with other herds? | 59.36 | 30.00 | 0.06 | 0.00 |
| A_7^3^ | How concerned are you about getting diseases from your animals if your livestock cross international borders? | 48.52 | 23.00 | 0.06 | 0.00 |
| A_8^3^ | How concerned are you about getting a disease if an animal bites you? | 37.92 | 32.00 | 0.02 | 0.22 |
| A_9^2^ | When mosquitoes are abundant during flooding, I am afraid of catching Rift Valley Fever disease. | 33.53 | 34.00 | 0.00 | 0.49 |
| A_10^2^ | When many animals are dying in my area, I am afraid of contracting a disease if I eat the meat of such animals. | 47.59 | 28.00 | 0.05 | 0.01 |
| A_12^3^ | How concerned are you about diseases spreading to humans during an outbreak of animal disease? | 21.20 | 24.00 | 0.00 | 0.63 |
| A_19^3^ | To what extent are you concerned that germs could become resistant when antimicrobials are used to treat humans? | 41.69 | 27.00 | 0.04 | 0.04 |
| A_21^3^ | How concerned are you about germs developing resistance when using antimicrobial drugs for yourself/family without a prescription? | 41.09 | 27.00 | 0.04 | 0.04 |
| A_22^3^ | How concerned are you about germs developing resistance when using antimicrobial drugs for your animals without a prescription? | 38.28 | 30.00 | 0.03 | 0.14 |
| A_27^2^ | The more antimicrobial dose I give to the animal, the higher the chance that the animal will recover. | 40.20 | 27.00 | 0.04 | 0.05 |
| A_28^2^ | When animals show improvement, there is no need to complete the entire prescribed course. | 34.82 | 26.00 | 0.03 | 0.12 |
| A_29^2^ | When humans show improvement, there is no need to complete the entire prescribed course. | 58.53 | 33.00 | 0.05 | 0.00 |
| A_33^1^ | How important do you think it is to wash your hands with soap after using a latrine or practicing open defecation? | 40.59 | 30.00 | 0.03 | 0.09 |
| A_34^1^ | How important is it for your health to wash hands before handling food? | 41.02 | 30.00 | 0.04 | 0.09 |
| A_35^1^ | How important is it for your health to wash hands after handling food? | 23.12 | 29.00 | 0.00 | 0.77 |
| A_36^2^ | Using soap and water kills germs much more effectively for hand cleaning compared to using only water. | 37.52 | 30.00 | 0.03 | 0.16 |
