# Supplementary material for: Development and validation of the OH-KAP survey for use with pastoral and other rural communities in Africa
Source: One Health Outlook. 2026 May 22;8:36. doi: 10.1186/s42522-026-00213-8 (PMC13404092; doi:10.1186/s42522-026-00213-8)

**Supplementary File 8.** This file provides the option characteristic curves (OCCs) and item information functions (IIFs) for the specific factors (S1-S5) of Attitude subscale from the bifactor IRT model. The main manuscript includes only the general One Health factor plots and the test information function (TIF); domain-specific plots are presented here for reference.


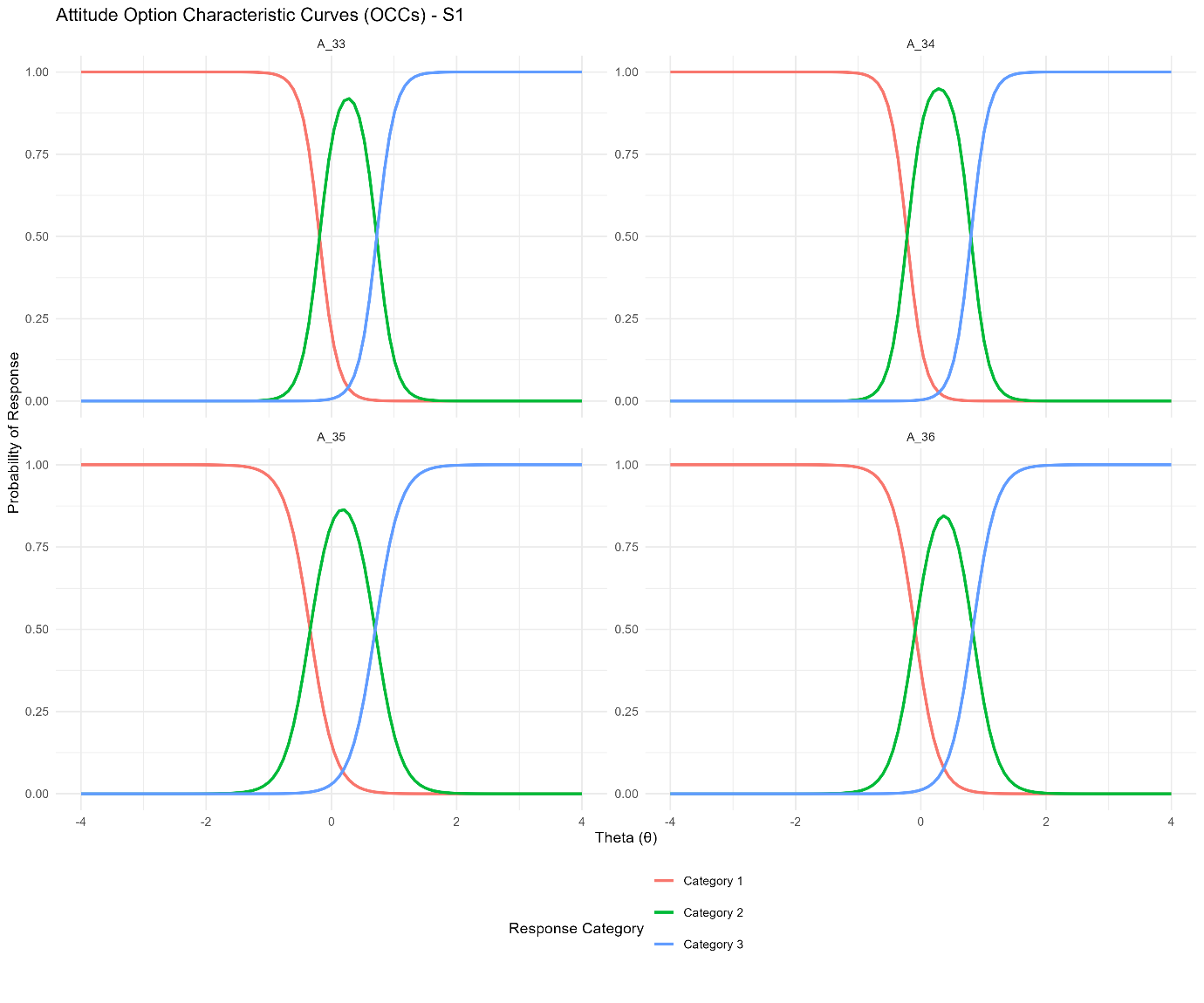


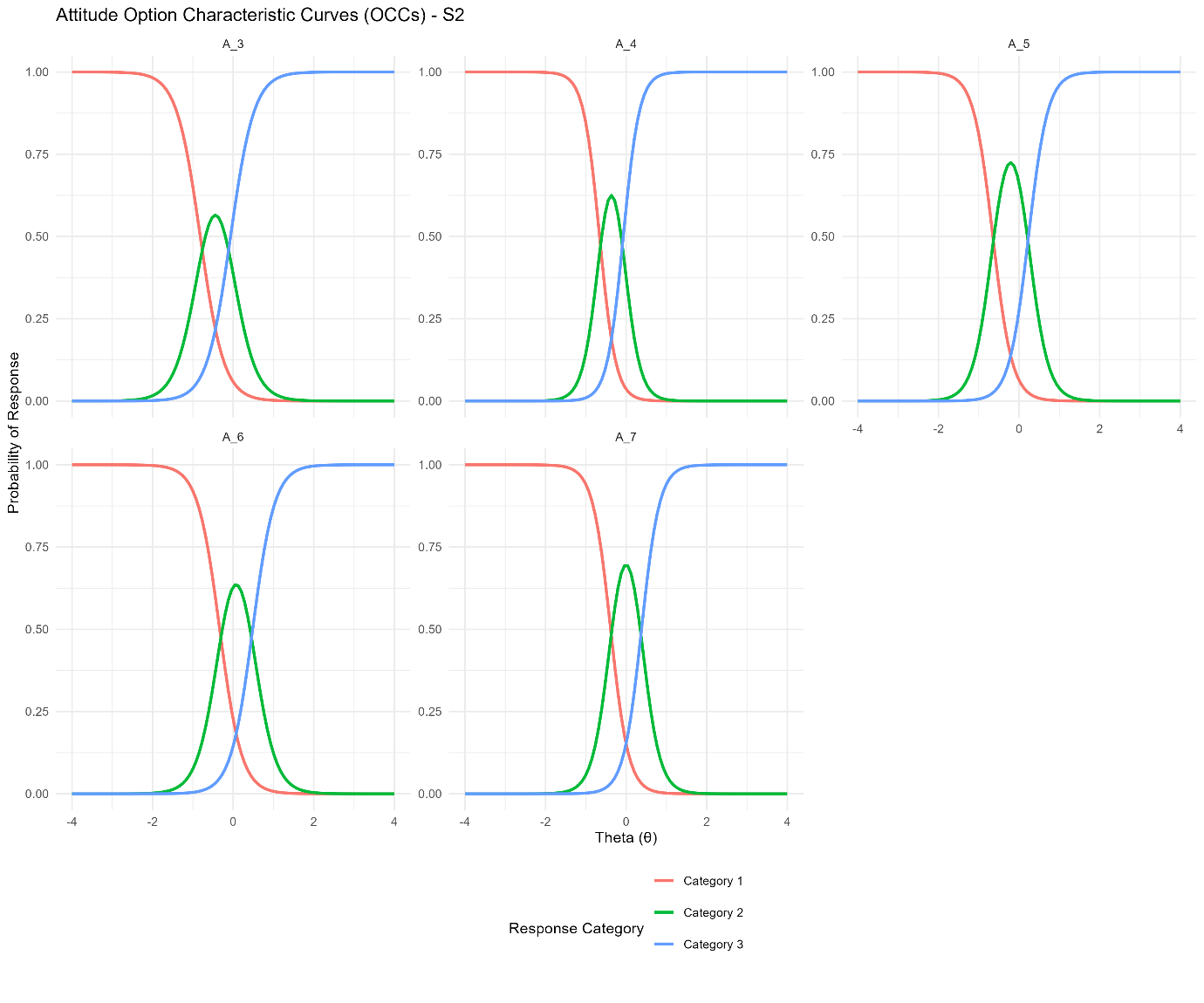

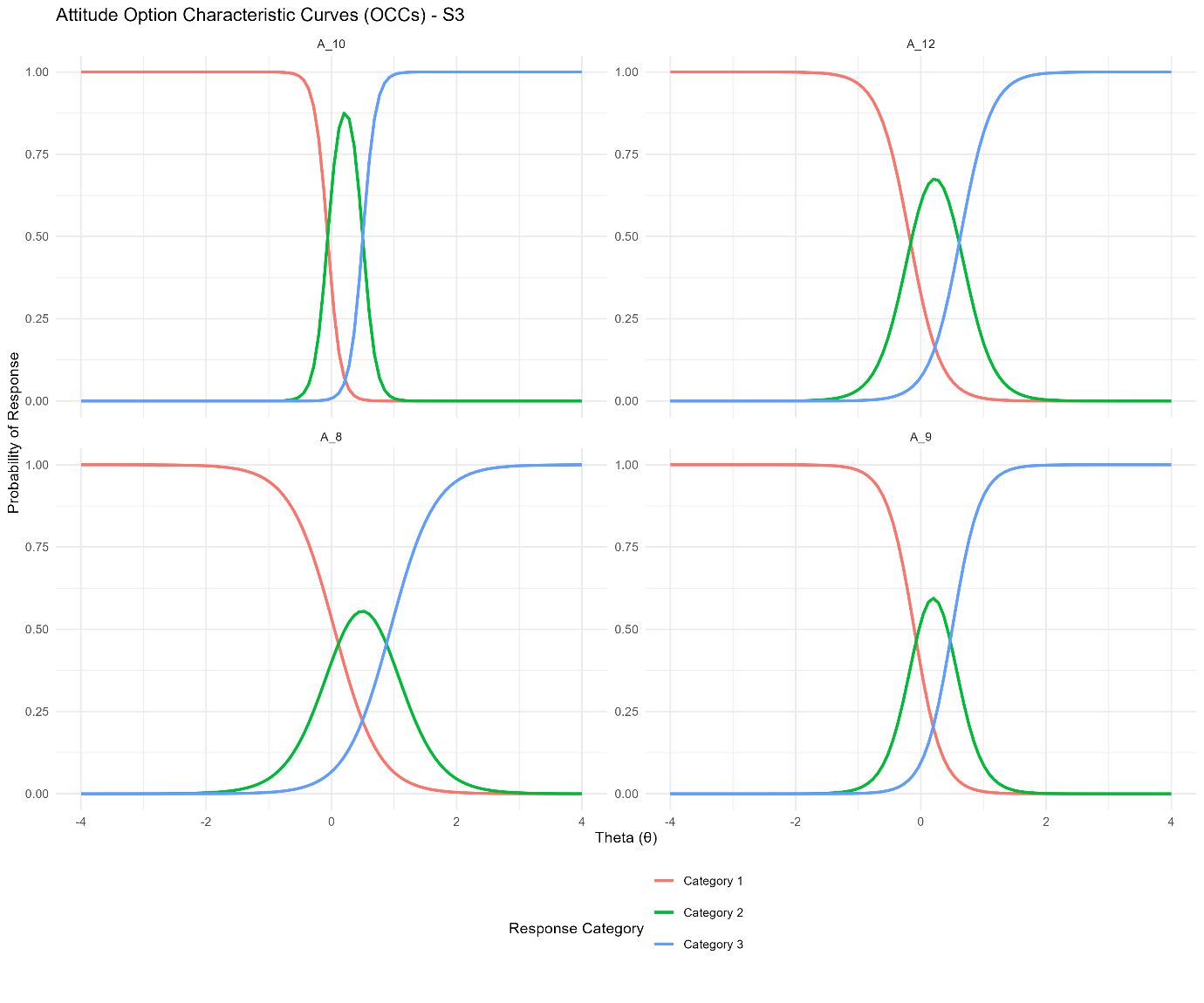

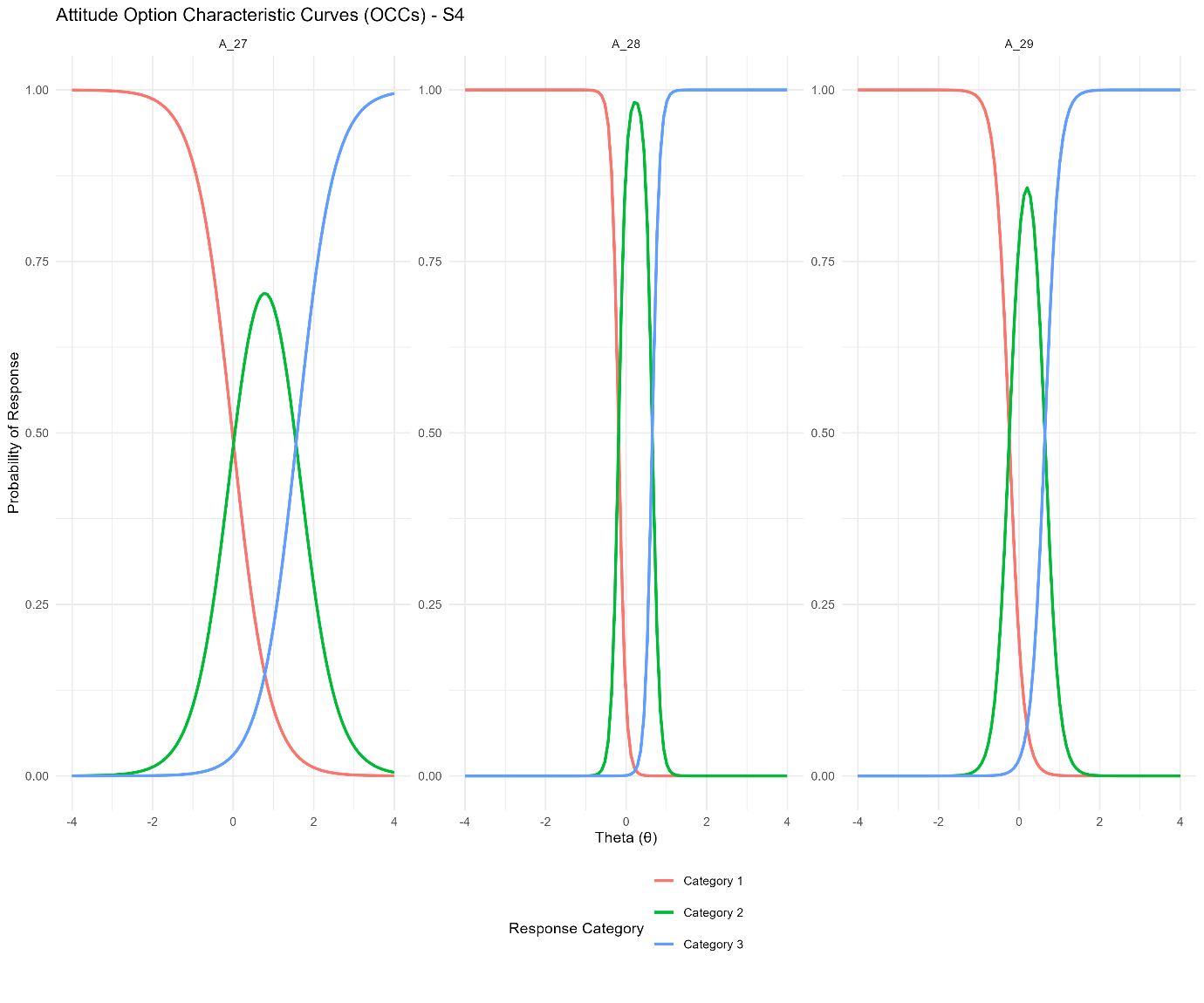

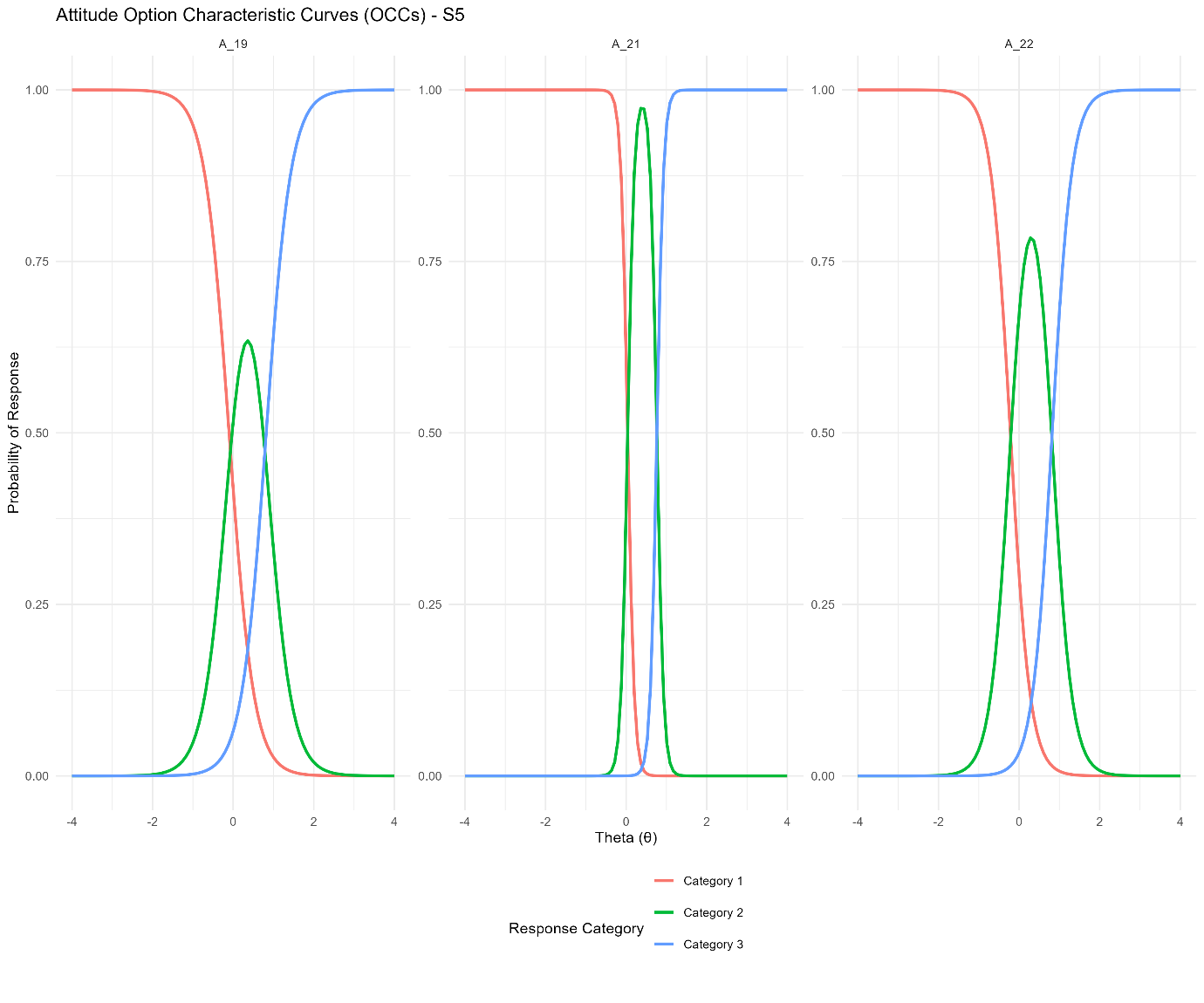


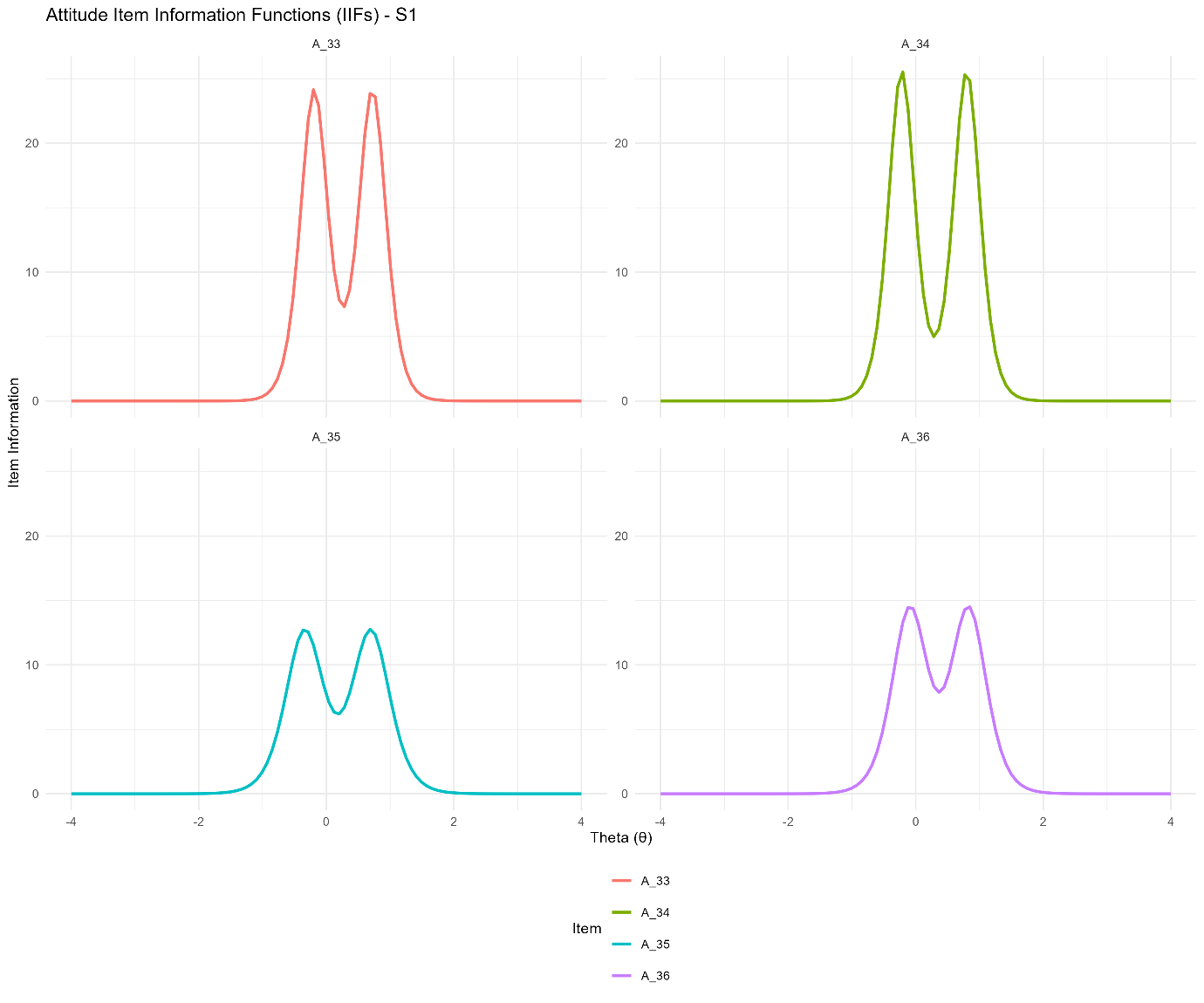

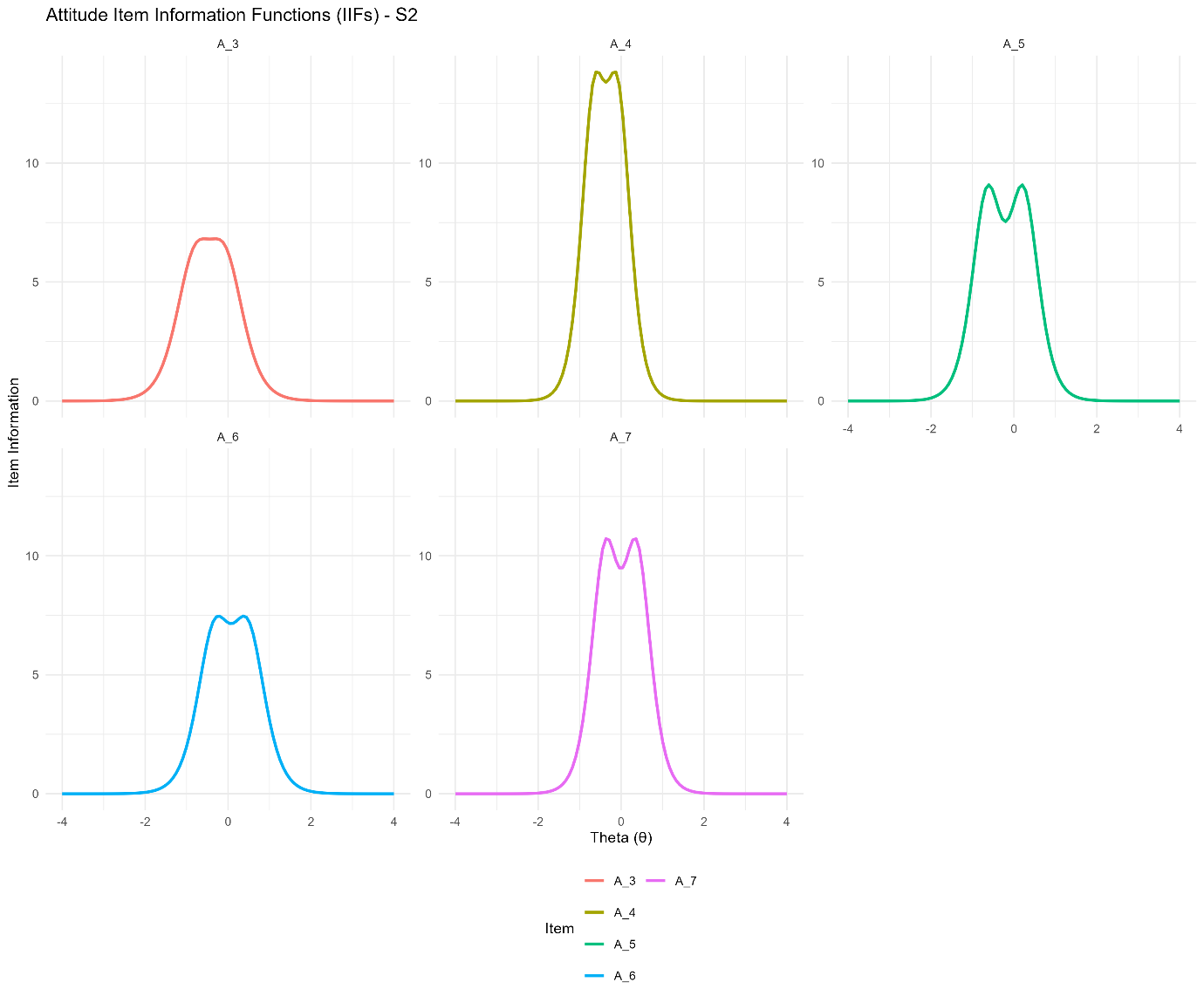

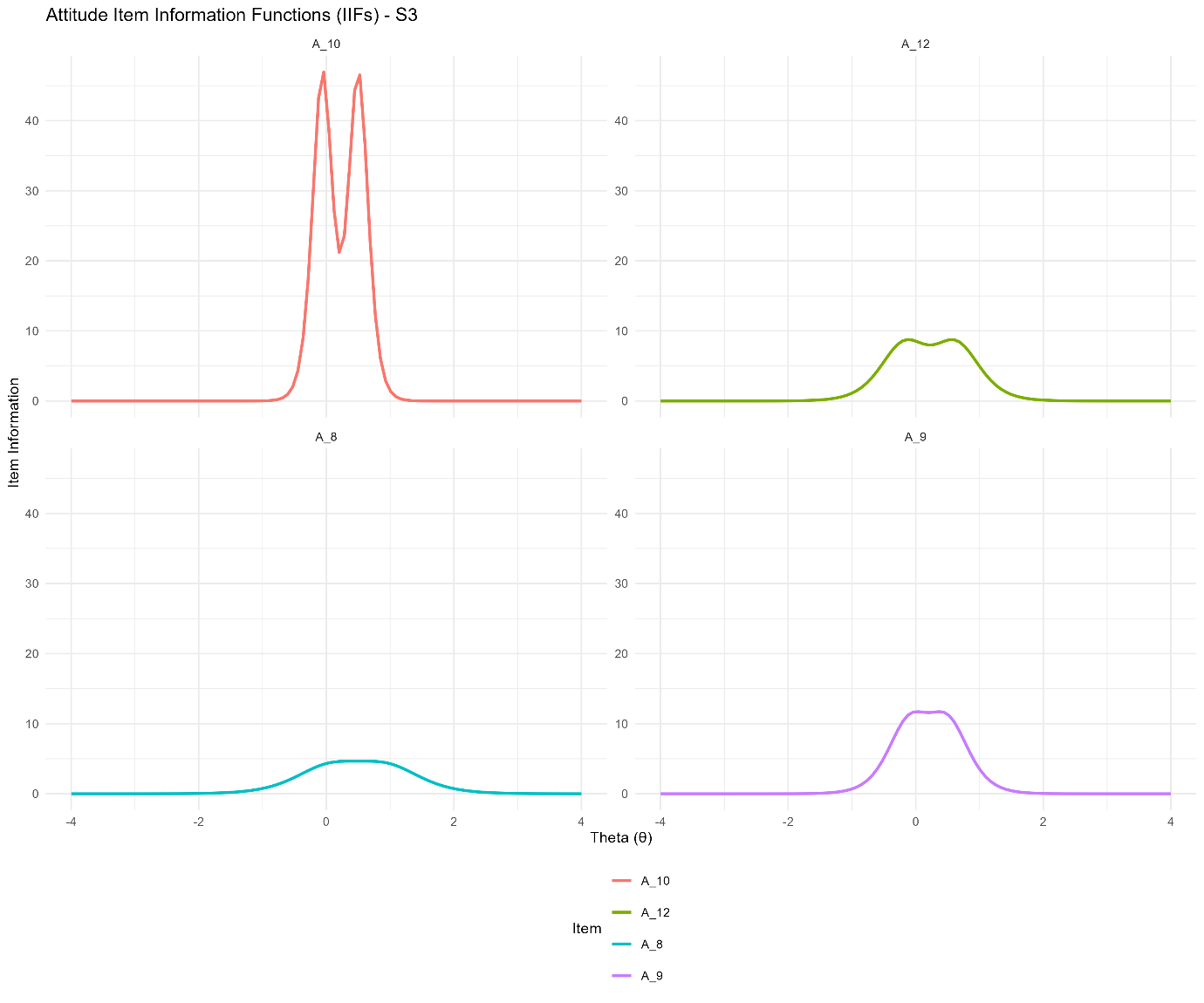

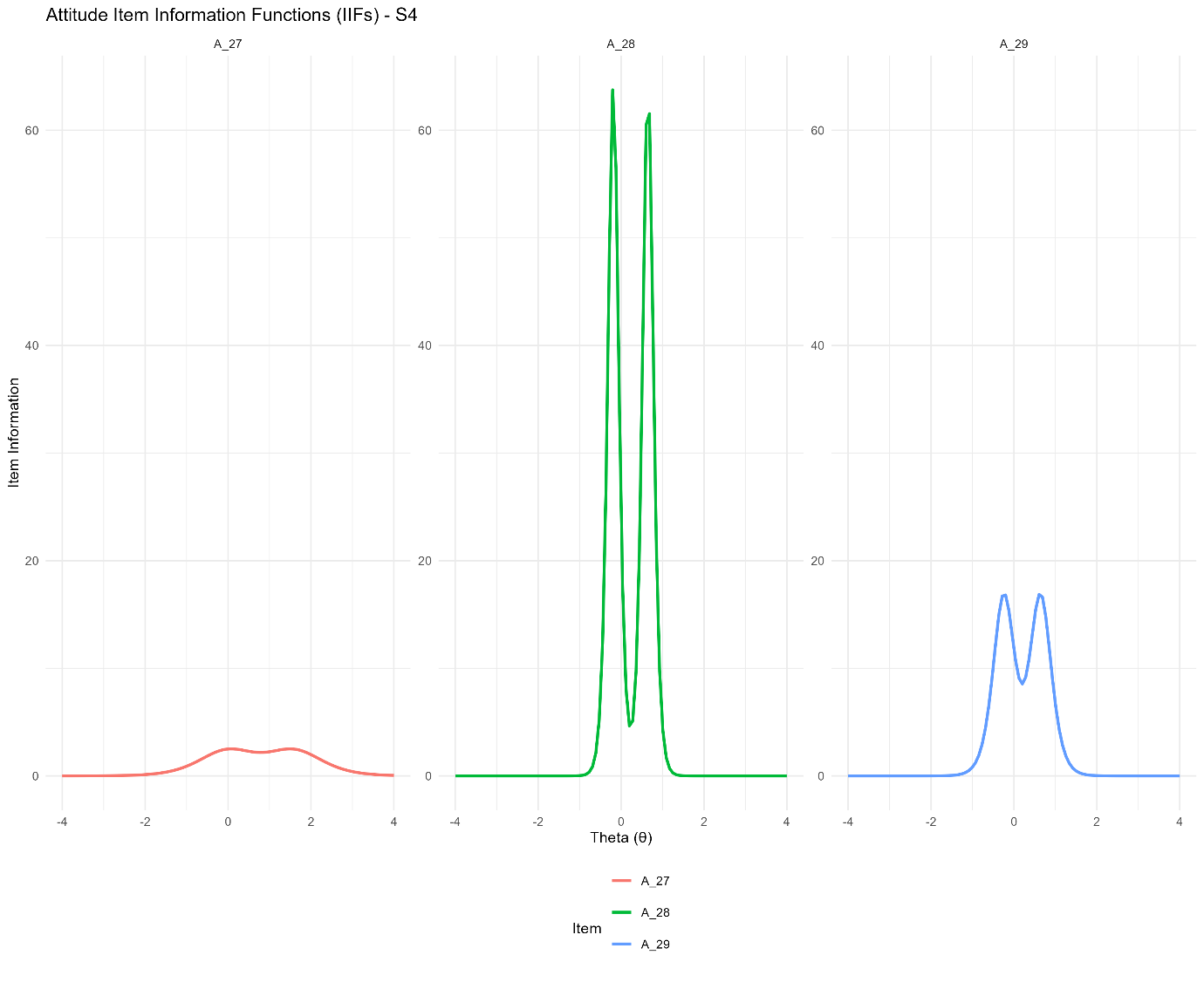

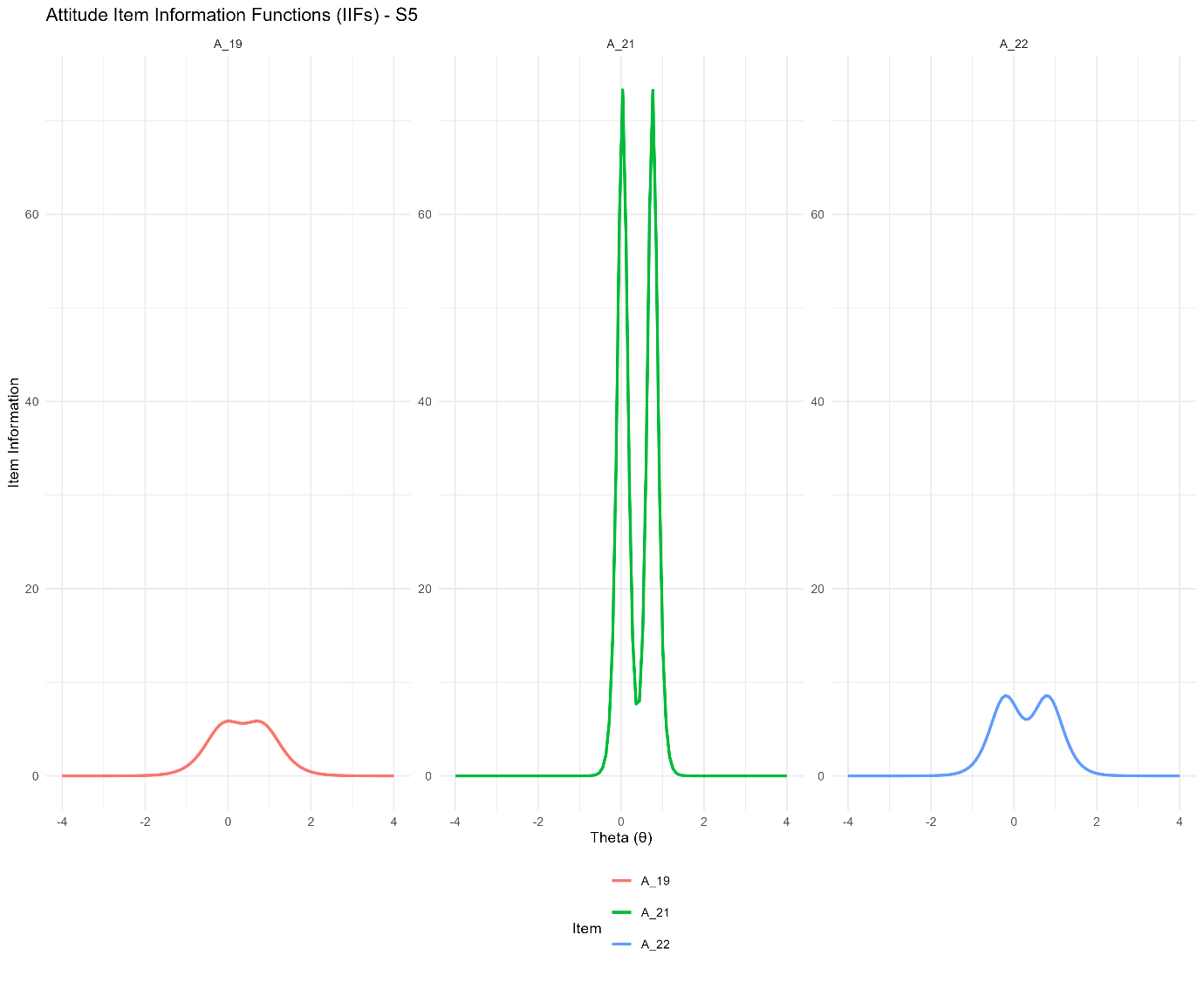

Supplement: Supplementary file 8 — Supplementary Material 8 [file 42522_2026_213_MOESM8_ESM.docx]
