# Supplementary material for: Development and validation of the OH-KAP survey for use with pastoral and other rural communities in Africa
Source: One Health Outlook. 2026 May 22;8:36. doi: 10.1186/s42522-026-00213-8 (PMC13404092; doi:10.1186/s42522-026-00213-8)
Supplement: Supplementary file 9 — Supplementary Material 9 [file 42522_2026_213_MOESM9_ESM.docx]

Supplementary File 4, Table 1. Implementation and scoring guide for the OH-KAP instrument

| **Why fixed parameters?**  Item parameters were estimated once during the original psychometric validation of OH-KAP. For subsequent applications (e.g., intervention studies, monitoring), these parameters are held fixed. This avoids re-calibration drift, allows direct comparability of scores across waves and studies, and reduces bias when sample sizes are modest or restricted. This approach follows best practice in applied IRT, where validated scales are scored with fixed parameters to maintain metric invariance. | |
| --- | --- |
| **Administration** | |
| **Population** | Adults (≥18 years) in pastoralist, agropastoralist, or mixed-farming communities. |
| **Mode** | Enumerator-administered, oral delivery in the local language. |
| **Training & ethics** | Enumerators trained in research ethics, neutral delivery, and local cultural norms. Written consent is preferred; thumbprint consent is acceptable for participants with limited literacy. |
| **Scoring approaches** | |
| **Knowledge** | - Response options: True, False, Don’t Know - Coding: 1 = correct; 0 = incorrect or DK - Scoring: θ estimated with a bifactor 2PL IRT model using fixed item parameters calibrated from OH-KAP Table 1. |
| **Attitudes** | - Response options: depending on item: Very Important / Somewhat Important / Not Important / Don’t know; Strongly Agree / Somewhat Agree / Don’t Agree / Don’t know; Very Concerned / Somewhat Concerned / Not Concerned / Don’t know; Very Risky / Somewhat Risky / Not Risky / Don’t know. - Coding: 0–3; DK = missing - Scoring: θ estimated with a bifactor GRM using fixed item parameters from the OH-KAP Table 2. |
| **Practices** | - - Response options: No/Never; Yes sometimes; Yes always; Don’t know   - Coding: 0; 1; 2; DK = missing   - Scoring: Dimension scores computed from EGA-validated item clusters with strength-based weights. |
| **Practical workflow** | 1. Collect responses with validated items, coded as specified. 2. Load the fixed item parameter files 3. Estimate Knowledge θ scores using *mirt::fscores* (method = "EAP"). 4. Estimate Attitude θ scores using *mirt::fscores* (method = "EAP"). 5. Compute Practice scores using provided EGA weights. 6. Analyze descriptively (e.g., distributions, medians) or analytically (e.g., univariate, multivariate). |
